# Investigating Causal Associations of Diet-Derived Circulating Antioxidants with the Risk of Digestive System Cancers: A Mendelian Randomization Study

**DOI:** 10.3390/nu14153237

**Published:** 2022-08-08

**Authors:** Xuening Zhang, Hao Zhao, Jinyu Man, Xiaolin Yin, Tongchao Zhang, Xiaorong Yang, Ming Lu

**Affiliations:** 1 Department of Epidemiology and Health Statistics, School of Public Health, Cheeloo College of Medicine, Shandong University, Jinan 250012, China; 2 Clinical Epidemiology Unit, Qilu Hospital of Shandong University, Jinan 250012, China; 3 Department of Medical Statistics and Epidemiology, School of Public Health, Sun Yat-sen University, Guangzhou 510080, China; 4 Clinical Research Center, Qilu Hospital of Shandong University, Jinan 250012, China

**Keywords:** antioxidant, ascorbate, retinol, digestive system cancers, mendelian randomization

## Abstract

Molecular mechanisms and observational studies have found that diet-derived antioxidants are associated with digestive system cancers, whereas there is a lack of causal evidence from randomized clinical trials. In this study, we aimed to assess the causality of these associations through a Mendelian randomization (MR) study. Single nucleotide polymorphisms of diet-derived circulating antioxidants (i.e., α- and γ-tocopherol, ascorbate, retinol, β-carotene, lycopene, and urate), accessed by absolute levels and relative metabolite concentrations, were used as genetic instruments. Summary statistics for digestive system cancers were obtained from the UK Biobank and FinnGen studies. Two-sample MR analyses were performed in each of the two outcome databases, followed by a meta-analysis. The inverse-variance weighted MR was adopted as the primary analysis. Five additional MR methods (likelihood-based MR, MR-Egger, weighted median, penalized weighted median, and MR-PRESSO) and replicate MR analyses for outcomes from different sources were used as sensitivity analyses. Genetically determined antioxidants were not significantly associated with five digestive system cancers, after correcting for multiple tests. However, we found suggestive evidence that absolute ascorbate levels were negatively associated with colon cancer in UK Biobank—the odds ratio (OR) per unit increase in ascorbate was 0.774 (95% confidence interval [CI] 0.608–0.985, *p* = 0.037), which was consistent with the results in FinnGen, and the combined OR was 0.764 (95% CI 0.623–0.936, *p* = 0.010). Likewise, higher absolute retinol levels suggestively reduced the pancreatic cancer risk in FinnGen—the OR per 10% unit increase in ln-transformed retinol was 0.705 (95% CI 0.529–0.940, *p* = 0.017), which was consistent with the results in UK Biobank and the combined OR was 0.747 (95% CI, 0.584–0.955, *p* = 0.020). Sensitivity analyses verified the above suggestive evidence. Our findings suggest that higher levels of antioxidants are unlikely to be a causal protective factor for most digestive system cancers, except for the suggestive protective effects of ascorbate on colon cancer and of retinol on pancreatic cancer.

## 1. Introduction

Digestive system cancers, most commonly including esophageal, stomach, colon, pancreatic, and liver cancers, are the leading cause of morbidity and mortality worldwide, accounting for some 4.2 million new cases and 3.1 million deaths each year [1]. The development of digestive system cancers is the result of the interaction of many factors, including lifestyle, metabolism, dietary, viral infection, and genetic factors. Apart from conventional risk factors, previous experimental evidence has also demonstrated that oxidative stress is one pathogenesis of digestive system cancers [2,3]. Previous studies have shown that the carcinogenic mechanism is that the continuous increase in reactive oxygen species (ROS) leads to oxidative stress, which induces the damage of macromolecules—such as DNA, proteins, and lipids—in cytosolic membranes and DNA mutations in nuclear membranes, resulting in malignant transformation [4,5]. Consequently, antioxidants would be of interest as targets for primary digestive system cancer prevention by scavenging the excess ROS to diminish oxidative stress-induced damage. In addition to the common mechanisms that reduce oxidative stress, previous preclinical studies have found specific mechanisms by which specific antioxidants may reduce the risk of digestive system cancers. Vitamin E may have a protective effect on the gastric mucosal injury induced by an H. pylori infection, through the inhibition of the accumulation of activated neutrophils [6]. Vitamin C can block the formation of nitrosamines, which is the strong carcinogen of digestive system cancers [7]. Vitamin A not only inhibits the carcinogenic effect of aflatoxin B, but also inhibits tumor growth on both exocrine and endocrine pancreatic cell lines [8,9,10]. Therefore, antioxidants may play a protective role in digestive system cancers. Specifically, diet-derived antioxidants are the most easily accessible and modifiable approach for consideration.

According to the possible protective effect mechanisms of antioxidants on the prevention of digestive system cancers, a large number of epidemiological studies have explored the relationship between antioxidants and digestive system cancers. Some observational studies have found that higher levels of vitamin E, vitamin C, and carotenoids in dietary components, supplements, or blood concentration are associated with a lower risk of esophageal cancer [11,12,13], stomach cancer [14], colon cancer [15,16], pancreatic cancer [17], and liver cancer [18]. However, other studies have shown that these antioxidants have little effect on digestive system cancers [19]. The inconsistent results reported in these observational studies may be caused by uncertain temporal relationships, insufficient sample sizes, short follow-up periods, the long-term nature of cancer progression or potential confounding factors. A previous randomized controlled trial (RCT) reported that antioxidant supplementation (vitamins A, C, and E) significantly reduced the recurrence of colorectal adenomas by 29% [20]. Another RCT has shown that vitamin E effectively and safely protects patients with stomach cancer from the occurrence of cisplatin neurotoxicity [21]. Overall, these RCTs focus more on the treatment and prognosis of cancer. Exploring the causal association between diet-derived antioxidants and the occurrence of digestive system cancers will have more public health significance for the primary prevention of these diseases. Unfortunately, given that the pathogenesis of the disease appears to be, perhaps, decades in length, RCTs are difficult to implement in this field. Therefore, it is necessary to improve the causal inference through other study designs.

Mendelian randomization (MR) uses genetic variants that are randomly allocated during conception, generally single-nucleotide polymorphisms (SNPs), as instrumental variables for exposure, which is similar to the random assignment in RCTs [22,23]. Therefore, MR analysis could provide a reliable estimation of the causal association between diet-derived antioxidants and the occurrence of digestive system cancers by minimizing measurement errors, confounding, and reverse causality [24]. With the development of sequencing technology, many large-scale genome-wide association studies’ (GWAS) data on diet-derived antioxidants and digestive system cancers have been published. This provides an opportunity for two-sample MR analysis using summary statistics from separate studies to substantially increase the statistical power by combining data from multiple sources. Previous studies based on this approach have explored the causal association of diet-derived antioxidants with Alzheimer’s disease, strokes, and coronary heart disease [25,26,27], while the causal association between diet-derived antioxidants and digestive system cancers has not been explored.

In this study, we used two-sample MR analyses to assess the causal associations between genetically predicted diet-derived absolute circulating antioxidants and their metabolites, with the risk of five digestive system cancers.

## 2. Materials and Methods

### 2.1. Overall Study Design

**Figure 1:**
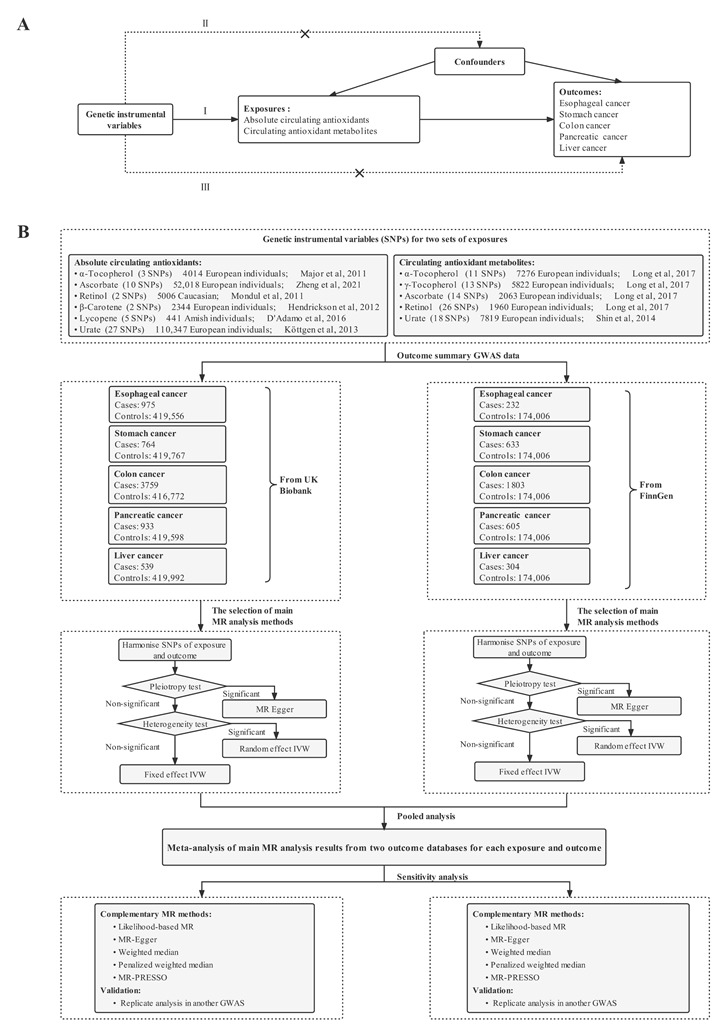
Schematic overview of the MR study design. (**A**) Principles of this MR study. There are three principal assumptions in MR design, as follows: (Ⅰ) the relevance assumption—the selected instrument is predictive of the exposure; (Ⅱ) the independence assumption—the instrument is not associated with any confounders of the exposure and outcome; and (Ⅲ) the exclusion-restriction assumption—the instrument is only associated with the outcome through the exposure. (**B**) Study design and framework of this research [29,30,31,32,33,34,35,36].

This study used two-sample MR analyses of summary-level genetic data to investigate whether diet-derived antioxidants, including α-and γ-tocopherol (i.e., vitamin E), ascorbate (i.e., vitamin C), retinol (i.e., vitamin A), β-carotene, lycopene, and urate, were causally associated with the risk of five digestive system cancers, including esophageal cancer, stomach cancer, colorectal cancer, pancreatic cancer, and liver cancer. For these diet-derived antioxidants, we examined the following two phenotypes: (1) absolute circulating antioxidants, measured as authentic absolute levels in the blood; and (2) circulating antioxidant metabolites, quantified as relative concentrations in plasma or serum. The principle of MR analysis and three assumptions of the instrumental variables are as follows: the relevance assumption, the independence assumption, and the exclusion-restriction assumption are presented in Figure 1A. [28]. The flowchart of MR analyses in this study is shown in Figure 1B.

### 2.2. Genetic Instruments Selection

For genetic instrumental variables of absolute circulating antioxidants, genetically determined α-tocopherol, ascorbate, retinol, β-carotene, lycopene, and urate were identified in the recent, large-scale GWAS’s data (*p* < 5 × 10^−8^, linkage disequilibrium [LD]: r^2^ = 0.001 and clump distance = 10,000 kb). Three independent, single nucleotide polymorphisms (SNPs) associated with α-tocopherol were obtained from the latest GWAS on 4014 individuals of European ancestry [29]. Ten independent SNPs associated with ascorbate were extracted from a recent GWAS of up to 52,018 individuals of European ancestry, according to the above threshold [30]. Two independent SNPs associated with retinol were identified from a GWAS of 5006 Caucasian participants, from two cohort studies [31]. Two independent SNPs associated with β-carotene were identified from a GWAS of 2344 participants in the Nurses′ Health Study [32]. Five independent SNPs associated with lycopene were identified from a GWAS involving 441 older Amish adults from Heredity and Phenotype Intervention Heart Study [33]. A total of 27 independent SNPs associated with urate were derived from a GWAS of up to 110,347 individuals of European ancestry, conducted by the Global Urate Genetics Consortium [34]. The summary information of the studies used for genetic instrumental variables extraction of absolute circulating antioxidants is shown in Supplementary Table S1.

For genetic instrumental variables of circulating antioxidant metabolites, genetically determined α-tocopherol, γ-tocopherol, ascorbate, retinol, and urate were identified in the recent large-scale GWAS’s data (*p* < 1 × 10^−5^, linkage disequilibrium [LD]: r^2^ = 0.001 and clump distance = 10,000 kb). Eleven SNPs for α-tocopherol (n = 7276), thirteen for γ-tocopherol (n = 5822), fourteen for ascorbate (n = 2063), and 18 SNPs for urate (n = 7819) were identified from 7824 adult participants of European ancestry, from two studies [35]. A total of 26 independent SNPs associated with retinol were identified from 1960 individuals of European ancestry [36].

Based on the PhennoScanner database (http://www.phenoscanner.medschl.cam.ac.uk/, accessed on 6 June 2022), the SNPs not reaching the genome-wide significance level associated with the confounders remained in the filtered genetic instrument [37]. Variance (R2) in MR study refers to the proportion of total variation in the exposure that is explained by the genetic instruments. R^2^ for each exposure was either derived from the original study or calculated based on the summary statistics of exposure by the following formula: R^2^ = (2 × EAF × (1 − EAF) × Beta^2^)/[(2 × EAF × (1 − EAF) × BETA^2^) + (2 × EAF × (1 − EAF) × N × SE^2^)]. Beta indicates the estimated genetic effect of SNP on exposure, EAF is effect allele frequency, SE is standard error of the estimated effect, and N is sample size. The F-statistic for each SNP was calculated by the following formula: BETA^2^/SE^2^. To avoid the risk of bias caused by weak instrumental variables, SNPs with F-statistics greater than 10 were retained as the final genetic instrument.

### 2.3. Outcome Data Sources

Summary-level genetic data for five digestive system cancers were obtained from the UK Biobank study and the FinnGen study. UK Biobank was a population-based cohort study that recruited more than 500,000 volunteers, aged 40–69 years, between 2006 and 2010. The second-round analysis of UK Biobank data from the Pan-UK Biobank project (https://pan.ukbb.broadinstitute.org/, accessed on 17 March 2022) was used in the present study [38]. In UK Biobank study, there were 975 cases of esophageal cancer and 419,556 non-cancer controls, 764 cases of stomach cancer and 419,767 non-cancer controls, 3759 cases of colon cancer and 416,772 non-cancer controls, 933 cases of pancreatic cancer and 419,598 non-cancer controls, and 539 cases of liver cancer and 419,992 non-cancer controls. FinnGen research project (https://www.finngen.fi/en, R6 released in 2022), combining genotype data from Finnish biobanks and digital health record data from Finnish health registries, provided a unique opportunity to study genetic variation in relation to disease trajectories in an isolated population. The FinnGen study ascertained 232 cases of esophageal cancer, 633 cases of stomach cancer, 1803 cases of colorectal cancer, 605 cases of pancreatic cancer, 304 cases of liver cancer, and 174,006 non-cancer controls, so far. If SNPs of instrument were not available in the outcome GWAS, the LDlink tool was used to identify proxy SNPs of European ancestry (r^2^ > 0.8) [39]. SNPs missing in the GWAS of outcome without appropriate proxy SNPs available were then excluded. The diagnostic information of five digestive system cancers in UK Biobank and FinnGen study is shown in Supplementary Table S2.

### 2.4. Statistical Analysis

The selection of main MR analysis methods is as follows: First, MR-Egger intercept test was performed to test whether there was the presence of potential pleiotropy [40]. If there was significant horizontal pleiotropy, the MR-Egger regression was used, otherwise the inverse-variance weighted (IVW) method was used. Notably, no significant horizontal pleiotropy was found in this study due to the rigorous screening of instrumental variables. Next, I^2^ statistic and *p*-value of Cochran′s Q-statistics test were used to assess heterogeneity [41]. If there was significant heterogeneity, the random-effect IVW model was used, otherwise the fixed-effect IVW model was used.

To further verify the robustness of our findings, some sensitivity analyses were performed. First, five complementary MR analysis methods were applied to help explain and verify causal inference, when applicable to different scenarios. The likelihood-based MR was considered the most accurate method to estimate causal effects when there was a continuous log-linear association between exposure and the risk of outcome (the number of SNPs more than 1) [42]. MR-Egger regression was conducted with bootstrapped standard errors to obtain pleiotropy-robust causal estimates (the number of SNPs more than 2). The weighted median can provide valid estimates if at least half of the weight comes from valid instrumental variables (the number of SNPs more than 2) [43]. The penalized weighted median method was implemented, which derives valid causal estimates even under conditions when invalid instruments are present (the number of SNPs more than 2) [43]. MR Pleiotropy RESidual Sum and Outlier (MR-PRESSO) was applied to detect and correct for horizontal polymorphisms by removing outliers (the number of SNPs more than 3) [44]. Second, the reproducibility of suggestive evidence discovery was further verified in other GWASs, including UK Biobank (European ancestry, self-reported colon cancer, 1494 cases, and 461,439 controls) and PanScan1 consortium (European ancestry, pancreatic cancer, 1896 cases, and 1939 controls).

All exposure-specific MR analyses were performed in each outcome database of UK Biobank and FinnGen study, and then meta-analysis was performed to generate the combined estimates for each exposure. The I^2^ statistic and *p*-value derived from Cochran′s Q-statistics test were assessed to the heterogeneity. If there was significant heterogeneity, the random effect model was selected, otherwise the fixed effect model was selected.

To account for multiple testing in our primary analyses, a Bonferroni-corrected threshold of *p* < 0.01 (α = 0.05/5 outcomes) was considered as significant evidence of associations, and a *p*-value between 0.01 and 0.05 was considered suggestive evidence of associations. All statistical analyses were performed using R Software (Version 4.1.0; R Foundation for Statistical Computing, Vienna, Austria). MR analyses were performed using the R-based “TwoSampleMR” package. Meta analyses were performed using the R-based “meta” package.

## 3. Results

### 3.1. Strength of Genetic Instruments

The summary information of the GWAS for diet-derived circulating antioxidants and their metabolites data are shown in Table 1. The genetic instruments of α-tocopherol, ascorbate, retinol, and urate were available both as absolute circulating antioxidants and metabolites. The variance explained by the genetic instruments ranged from 1.7% to 30.1% for absolute antioxidant levels (all F statistic > 10) and from 6.8% to 21.7% for antioxidant metabolites (all F statistic > 10). The raw data information on the effect estimation of the associations of selected SNPs with antioxidants and with digestive system cancers are given in Supplementary Tables S3 and S4.

### 3.2. Absolute Circulating Antioxidants and Digestive System Cancers

**Figure 2:**
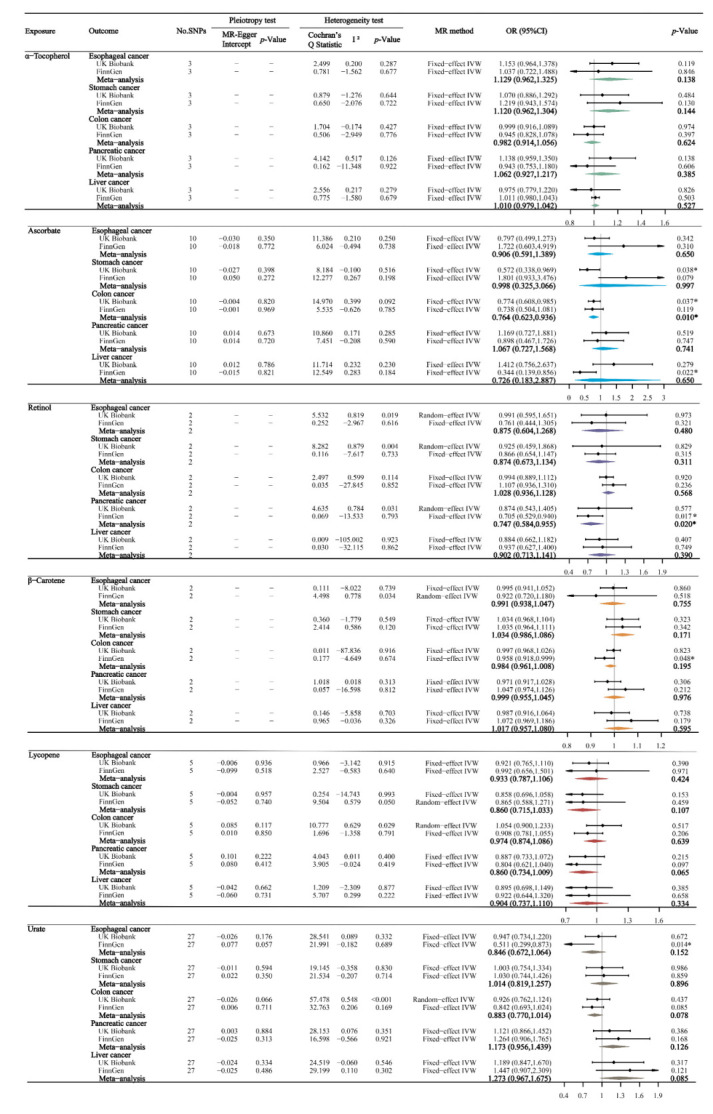
The main MR analyses results of the causal effects of absolute circulating antioxidant levels on five digestive system cancers. If there was significant heterogeneity (*p*-value of Cochran′s Q Statistic < 0.05) we used the random-effect IVW model, otherwise we used the fixed-effect IVW model. The odds ratios were scaled per 10% unit increase in log-transformed α-tocopherol values, per unit increase in ascorbate, per 10% unit increase in ln-transformed retinol, per 10% unit increase in ln-transformed β-carotene, per unit increase in lycopene, and per unit increase in urate. Statistical significance was defined as Bonferroni-corrected threshold of *p*-value < 0.01 (0.05/5), and *p*-value between 0.01 and 0.05 was considered suggestive evidence (*) of associations.

Figure 2 shows the primary results of the MR estimates for absolute circulating antioxidants. The MR-Egger intercept test found no significant horizontal pleiotropy for all outcomes, with *p*-values ranging from 0.07 to 0.96. In the main results by IVW methods, we did not find any significant associations between absolute blood antioxidant levels and the risk of the five digestive system cancers, after Bonferroni correction (all *p* > 0.01). However, we found suggestive evidence that genetically determined, higher absolute ascorbate levels (per unit in log-transformed) were associated with a reduced risk of stomach cancer and colon cancer in the UK Biobank study. Furthermore, there were suggestive negative causalities between absolute urate and esophageal cancer, β-Carotene and colon cancer, retinol and pancreatic cancer, and ascorbate and liver cancer in the FinnGen study.

After combining the UK Biobank and FinnGen studies, a meta-analysis showed that the absolute ascorbate levels had a suggestive protective effect against colon cancer (OR 0.764; 95% confidence interval [CI] 0.623–0.936, *p* = 0.010), and the association direction and magnitude in the FinnGen study (OR 0.738; 95% CI, 0.504, 1.081, *p* = 0.119) remained consistent with that in the UK Biobank study (OR 0.774, 95% CI 0.608–0.985, *p* = 0.037). Similarly, the meta-analysis also showed that higher retinol levels (per unit in log-transformed) suggestively reduced the risks of pancreatic cancer (OR 0.747; 95% CI 0.584–0.955, *p* = 0.020), and the association direction and magnitude in the UK Biobank study (OR 0.874; 95% CI 0.543–1.405, *p* = 0.577) remained consistent with that in the FinnGen study (OR 0.705; 95% CI 0.529–0.940, *p* = 0.017).

### 3.3. Circulating Antioxidant Metabolites and Digestive System Cancers

**Figure 3:**
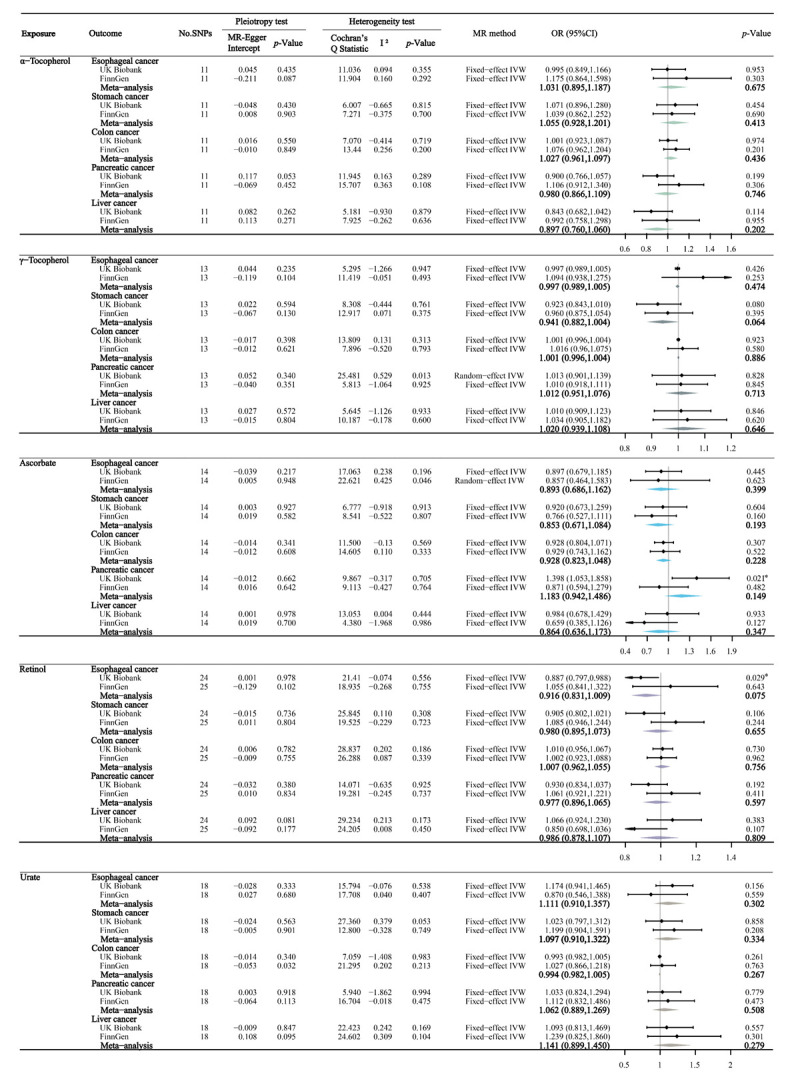
The main MR analyses results of the causal effects of circulating antioxidant metabolites on five digestive system cancers. If there was significant heterogeneity (*p*-value of Cochran′s Q Statistic < 0.05), we used the random-effect IVW model, otherwise we used the fixed-effect IVW model. The odds ratios were scaled per 10% unit increase in log-transformed α-tocopherol, γ-tocopherol, and urate values, and per unit increase in log-transformed ascorbate and retinol values. Statistical significance was defined as Bonferroni-corrected threshold of *p*-value < 0.01 (0.05/5), and *p*-value between 0.01 and 0.05 was considered suggestive evidence (*) of associations.

Figure 3 shows the primary results of the MR estimates for circulating antioxidant metabolites. The MR-Egger intercept test also found no significant horizontal pleiotropy for all outcomes, with *p*-values ranging from 0.06 to 0.94. Similar to the findings from the MR analyses of absolute antioxidants, there was no significant evidence that genetically determined circulating antioxidant metabolites were significantly associated with the risk of the five digestive system cancers, after Bonferroni correction (all *p* > 0.01). However, we found a suggestively positive association between circulating ascorbate metabolites and pancreatic cancer (OR 1.398; 95% CI 1.053–1.858, *p* = 0.021) and a suggestively negative association between retinol and esophageal cancer (OR 0.887; 95% CI 0.797–0.988, *p* = 0.029). After combining the UK Biobank and FinnGen studies, a meta-analysis showed that none of these associations had statistical significance.

### 3.4. Sensitivity Analysis

**Figure 4:**
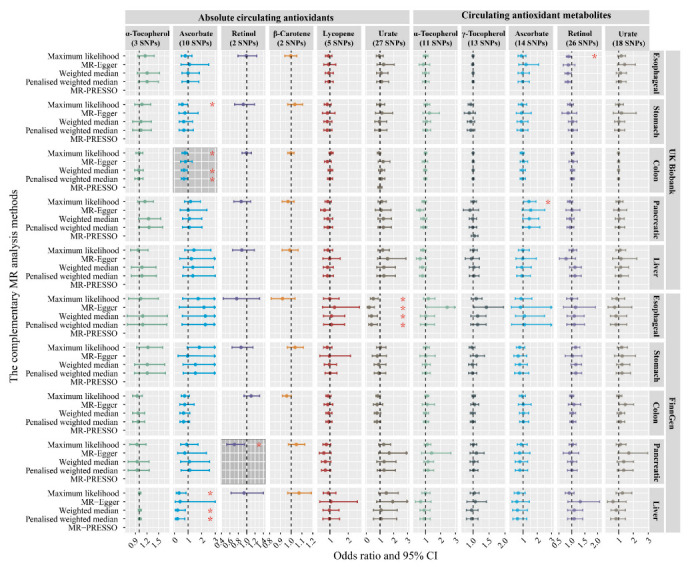
The complementary MR analyses results of the causal effects of diet-derived circulating antioxidants on five digestive system cancers. The MR-Egger, weighted median, and penalized weighted median required the number of SNPs in the instrumental variable > 2, and MR-Egger method could not accurately estimate due to collinearity in MR analyses for α-tocopherols. The MR-PRESSO requires the number of SNPs in the instrument variable > 3. If the MR-PRESSO global test did not identify significant outliers in the genetic instrument, the MR-PRESSO did not require correction. The error bars indicate 95% CIs. Statistical significance was defined as Bonferroni-corrected threshold of *p*-value < 0.01 (0.05/5), and *p*-value between 0.01 and 0.05 was considered suggestive evidence (*) of associations. Shaded areas represent suggestive evidence from meta-analysis, combining UK Biobank and FinnGen.

Figure 4 and Supplementary Tables S5 and S6 show the results through a variety of complementary MR analyses. For the MR results of the SNPs with a number more than two, complementary MR analysis methods proved that there were suggestive associations between absolute urate and esophageal cancer, absolute ascorbate and colon cancer, and absolute ascorbate and liver cancer. In particular, for the validation of the suggestive evidence from the meta-analysis, three supplementary MR analysis results, including maximum likelihood, weighted-median estimator, and penalized weighted median MR analysis methods, indicated suggestive evidence that genetically determined higher absolute ascorbate reduced the risk of colon cancer (OR, 0.771 [95% CI, 0.605–0.983], *p* = 0.036; 0.699 [95% CI, 0.512–0.953], *p* = 0.024; and 0.699 [95% CI, 0.510–0.958], *p* = 0.026). For most outcomes, the effect size and direction of the MR-Egger method were consistent with the IVW method, though with low precision. The MR-PRESSO method identified outlier SNPs for absolute urate on colon cancer and for γ-Tocopherol metabolites on pancreatic cancer in the UK Biobank study. MR analyses after removing outliers showed that the OR estimates did also not change significantly. No outlier SNPs were identified in the MR-PRESSO analysis for the other outcomes.

For the MR analyses with two SNPs, the results of the likelihood ratio method were basically consistent with the main analysis results. Notably, we found that a genetically determined higher level of absolute retinol was associated with a reduced risk of pancreatic cancer in the FinnGen study (OR, 0.705; 95% CI, 0.525–0.946; *p* = 0.020).

To further verify the discovery of suggestive evidence, we found that genetically determined higher absolute ascorbate levels reduced the risk of colon cancer from another GWAS of self-reported colorectal cancer (*p* = 0.036, Supplementary Table S7). In addition, we found that the negative association between absolute retinol and pancreatic cancer from the GWAS of the PanScan1 consortium was not statistically significant, possibly due to the small sample size (Supplementary Table S7).

## 4. Discussion

In the present study, we conducted the first comprehensive two-sample MR study to investigate whether there is a potential causal association between genetically determined, diet-derived circulating antioxidants and the risk of five digestive system cancers. Genetic variants were proxied as instrumental variables for absolute circulating antioxidant levels and their metabolite concentrations. Our findings indicate that genetically determined diet-derived antioxidants were not significantly associated with the five digestive system cancers. However, we found suggestive evidence from the meta-analysis combining the UK Biobank and FinnGen studies that genetically determined, higher absolute circulating ascorbate and retinol levels reduced the risk of colon cancer and pancreatic cancer, respectively.

Ascorbate, also known as vitamin C, is a powerful antioxidant that reduces the oxidative stress from ascorbate peroxidase. It cannot be synthesized by itself and must be ingested through food and medicine. Dietary source vitamin C is widely found in fresh fruits and vegetables, which is usually absorbed by the intestine and only a small amount is absorbed by the stomach. The European Prospective Investigation into Cancer and Nutrition study, involving 898 colon cancer cases and 1399 controls, suggested that dietary vitamin C was inversely associated with the risk of distal colon cancer [OR (per 63 g/day): 0.83, 95% CI: 0.70, 0.97] [45]. Another prospective cohort study, the NIH-AARP Diet and Health Study, indicated that a high intake of vitamin C during the ages 40–61 years was inversely associated with colon cancer after the age of 50 years (HR: 0.83, 95% CI: 0.72, 0.95) [46]. A meta-analysis of prospective cohort studies reported that high (>600 mg/day) versus low (≤100 mg/day) vitamin C intake was associated with a 19% lower risk of colon cancer [16]. Previous RCT studies have reported that a high dose of intravenous ascorbate acid could decrease the pain and the chemotherapy-related side effects for colon cancer patients [47,48]. The above studies may be considered to support our findings of the negative associations between genetically predicted ascorbate and colon cancer. Furthermore, although previous MR studies reported an inverse association between ascorbate and colon cancer, no suggestive evidence or significant causal association was found, which may be due to the use of non-independent SNPs as instrumental variables to attenuate the protective effect of ascorbate [49]. We found that previous biological mechanism studies could partially explain this causal relationship. First, ascorbate reduces the concentration of nitroso compounds by competing with amine to bind nitrate, block the nitrosylation reaction, promote the decomposition of nitrosamine, and, finally, inhibit the carcinogenic effect of N-nitroso compounds [50]. Second, vitamin C selectively kills KRAS and BRAF mutant colorectal cancer cells by targeting glyceraldehyde 3-phosphate dehydrogenase [51]. Third, vitamin C activates pyruvate dehydrogenase, then modulates the TCA cycle and the mitochondrial metabolism in KRAS mutant colon cancer [52].

Retinol is one of the most biologically active forms of vitamin A and is a biochemical index of vitamin A status in the human body [31]. Twenty years ago, a few case-control studies on pancreatic cancer, which were based on dietary questionnaires, did not yield consistent conclusions (ORs ranged from 0.53 to 1.70), possibly due to inconsistent dietary questionnaires and the small sample sizes [53]. Based on these low-quality studies, the meta-analysis also reported no significant association between retinol and pancreatic cancer [53]. However, recent studies have reported that patients with pancreatic cancer have reduced serum levels of the retinol-binding protein (case: 3.5 mg/100 mL vs. control: 5.6 mg/100 mL), a transporter of vitamins in the blood, reflecting vitamin A (retinol) deficiency [54]. Similar to our study, we also found that higher levels of retinol slightly reduced the pancreatic cancer risk. In addition, a multicenter phase II study supported the efficacy and safety of immunotherapy, including 13-cis-retinoic acid and interleukin 2 in locally advanced pancreatic cancer patients [55]. The protective mechanisms of retinol (vitamin A) on pancreatic cancer are as follows: First, retinoids could cause apoptosis in pancreatic cancer cells and, thus, suppress pancreatic cancer growth via the activation of retinoic acid receptor-gamma, suggesting that vitamin A and its metabolites may play a protective role against pancreatic cancer [56]. Second, ATRA, an active metabolite of vitamin A, may help shut down activated pancreatic stellate cells and prevent the formation of connective tissue around tumors, thereby having an anti-tumor effect [57]. Third, retinoic acid could inhibit pancreatic cancer cell migration and epithelial-mesenchymal transition by decreasing the expression of interleukin 6 in cancer-associated fibroblast cells, suggesting that retinoids could be applied to prevention or therapy in the recurrence and metastasis of pancreatic cancer [58].

The above observational studies, RCT, and molecular mechanism studies can support our findings. However, the effects of genetic susceptibility are lifelong, whereas the effects of antioxidant supplementation may only last up to the trial period. Considering that short-term supplementation therapy may not alter long-term risk—similar to the results of the nutritional intervention studies conducted by Wang et al. [59]—slight exposure throughout life would have greater potential biological effects than a temporary higher dose of supplements. Therefore, from the perspective of disease prevention, we encourage the long-term stable maintenance of antioxidants (especially ascorbate and retinol) in the body at slightly high levels, within the normal range, through dietary intake, which may help reduce the risk of colon cancer and pancreatic cancer.

Our study has several advantages. First, the MR design of two independent samples, based on genetic instrumental variables, reduced the possibility of subjects being exposed to unnecessary risks and hazards in the RCT study and supplemented the genetic theoretical basis of dietary antioxidants and digestive system cancers. Second, we used two independent sets of instrumental variables, including the absolute blood levels of antioxidants and the corresponding circulating metabolite concentrations. The effect direction of the antioxidants on the cancers was generally consistent in both sets, suggesting that the measurements were reliable. Third, this study examined the associations in two independent populations and performed a meta-analysis. The consistency of the results demonstrates the reliability of our findings. The combination of two large databases increased the sample size of this study and, thus, enhanced our ability to detect associations. Finally, our study performed a series of sensitivity analyses, which showed that the results obtained by other methods were consistent with the main IVW method. This, again, verifies the robustness of our results. The pleiotropic assessment confirmed that our findings were not biased by pleiotropic effects.

Inevitably, there were several limitations in our study. First, our analyses were based on published summary data rather than individual data, therefore, we were unable to test nonlinear causal relationships between antioxidant levels and the risk of developing the selected digestive system cancers. Similarly, due to the lack of individual cancer histological subtype data, the associations between the different cancer histological subtypes and the antioxidants could not be explored. Second, although an inherent limitation of MR analysis is that there may be potential polymorphic effects, the MR-Egger intercept test in our study showed no significant pleiotropy, and no SNPs significantly associated with confounding were detected in the PhennoScanner database [37], indicating that horizontal pleiotropy was unlikely to exist. Third, although we only observed a slight protective effect in vitamin C and retinol and not in other dietary sources of antioxidants, we cannot completely rule out the possibility that the effect size of tocopherol, β-carotene, lycopene, and urate was too small to be identified. Fourth, the relatively low power to detect the suggestive effects of antioxidants on the risk of digestive system cancers, due to the small number of reliable genetic instruments available for the antioxidants of interest and due to the limited number of cases of digestive system cancers in the UK Biobank study. This could be improved in the future as a larger sample GWAS for both the exposure and outcome becomes available. Finally, due to the limited availability of GWASs of antioxidants, multivariable MR analyses of the combined effects of multiple antioxidants on specific cancers could not be performed.

## 5. Conclusions

In conclusion, the present study showed that diet-derived antioxidants were not significantly associated with the five digestive system cancers. However, we found suggestive evidence that genetically predicted, dietary-derived higher absolute ascorbate and retinol concentrations were associated with a marginally reduced risk of colon cancer and pancreatic cancer, respectively. Future MR studies with larger replication samples of genetic data on the corresponding cancers and larger GWASs of circulating antioxidants are needed to validate our findings.

## Figures and Tables

**Table 1:** The summary of instrumental variables for diet-derived absolute circulating antioxidants and antioxidant metabolites.

Trait	Sample Size	*p*-Value	LD	No. of SNPs	Explained Variance (R^2^, %) *	Unit	PMID
Absolute circulating antioxidants							
α-Tocopherol	4014	5 × 10^−8^	0.001	3	1.7	mg/L in log-transformed scale	21729881
Ascorbate	52,018	5 × 10^−8^	0.001	10	1.7	µmol/L	33203707
Retinol	5006	5 × 10^−8^	0.001	2	2.3	µg/L in ln-transformed scale	21878437
β-Carotene	2344	5 × 10^−8^	0.001	2	4.8	µg/L in ln-transformed scale	23134893
Lycopene	441	5 × 10^−8^	0.001	5	30.1	µg/dL	26861389
Urate	110,347	5 × 10^−8^	0.001	27	3.7	mg/dL	23263486
Circulating antioxidant metabolites							
α-Tocopherol	7725	1 × 10^−5^	0.001	11	6.8	log10-transfomed metabolite concentration	24816252
γ-Tocopherol	6226	1 × 10^−5^	0.001	13	9.8	log10-transfomed metabolite concentration	24816252
Ascorbate	2085	1 × 10^−5^	0.001	14	21.7	log10-transfomed metabolite concentration	24816252
Retinol	1960	1 × 10^−5^	0.001	26	20.6	log10-transfomed metabolite concentration	28263315
Urate	7819	1 × 10^−5^	0.001	18	11.4	log10-transfomed metabolite concentration	24816252

LD—linkage disequilibrium. *—variance explained (R^2^) were either derived from the original study or formula calculation.

## Data Availability

The raw data used to support the findings of this study are included within the supplementary material. All summary statistics for the two-sample Mendelian randomization are available online, from each genome-wide association study consortia.

## Supplementary Materials

The following supporting information can be downloaded at: https://www.mdpi.com/article/10.3390/nu14153237/s1. Supplementary Table S1: The summary information of the studies used for genetic instrumental variable extraction of absolute circulating antioxidants. Supplementary Table S2: The diagnostic information of five digestive system cancers in UK Biobank and FinnGen. Supplementary Table S3: The causal effect estimates of the associations between genetic instrumental variables for absolute circulating antioxidants and the risk of five digestive system cancers. Supplementary Table S4: The causal effect estimates of the associations between genetic instrumental variables for circulating antioxidant metabolites and risk of five digestive system cancers. Supplementary Table S5: The complementary MR analyses results of the causal effects of absolute circulating antioxidants on five digestive system cancers. Supplementary Table S6: The complementary MR analyses results of the causal effects of circulating antioxidant metabolites on five digestive system cancers. Supplementary Table S7: For the outcomes from other GWAS studies, the sensitivity MR analyses results of the causal effects of absolute ascorbate and retinol level on colon and pancreatic cancer.
