# Supplementary material for: Investigating Causal Associations of Diet-Derived Circulating Antioxidants with the Risk of Digestive System Cancers: A Mendelian Randomization Study
Source: Nutrients. 2022 Aug 8;14(15):3237. doi: 10.3390/nu14153237 (PMC9370260; doi:10.3390/nu14153237)
Supplement: Supplementary file 1 [file nutrients-14-03237-s001.zip › nutrients-1782722-supplementary.pdf]

Supplementary Tables

Table S1 The summary information of the studies used for genetic instrumental variables extraction of absolute circulating antioxidants. 2

Table S2 The diagnostic information of five digestive system cancers in UK Biobank and FinnGen. 3

Table S3 The causal effect estimates of the associations between genetic instrumental variables for absolute circulating antioxidants and risk of five digestive system cancers. 4

Table S4 The causal effect estimates of the associations between genetic instrumental variables for circulating antioxidant metabolites and risk of five digestive system cancers. 8

Table S5 The complementary MR analyses results of the causal effects of absolute circulating antioxidants on five digestive system cancers. 15

Table S6 The complementary MR analyses results of the causal effects of circulating antioxidant metabolites on five digestive system cancers. 19

Table S7 For the outcomes from other GWAS studies, the sensitivity MR analyses results of the causal effects of absolute ascorbate and retinol level on colon and pancreatic cancer. 23

Table S1 The summary information of the studies used for genetic instrumental variables extraction of absolute circulating antioxidants.

Absolute circulating antioxidants	Sample size	Age	Sex (male)	Antioxidant concentration
**α-Tocopherol (mg/L)** [1]				
ATBC	4,014	58.1±5.0	100%	11.9 ±3.4
**Ascorbate (μmol/L)** [2]				
Fenland GWAS	1,349	45±7	44%	66.2±21.3
Fenland UKBB	8,391	49±7	47%	68.6±21.5
Fenland Core Exome	1,031	51±7	45%	68.3±21.8
InterAct subcohort GWAS	3,521	51±9	35%	42.8±19
InterAct subcohort core-exome	6,504	53±9	38%	42.9±19.1
InterAct non-subcohort GWAS	2,944	55±8	48%	36.4±17.6
InterAct non-subcohort core-exome	3,872	56±7	52%	36.5±18.9
EPIC-Norfolk GWAS	16,756	59±9	47%	53.8±20.2
EPIC-CVD subcohort	885	53±12	41%	41.0±21.0
EPIC-CVD non-subcohort	6,765	57±8	55%	37.9±20.8
**Retinol (μg/dL)** [3]				
ATBC	4,014	58.1±5.0	100%	572 (796-654)
PLCO	992	64.6 ± 4.9	100%	672 (562-794)
**β-Carotene (μg/L)** [4]				
NHS	2,344	58.8±6.4	0%	303±258
**Lycopene (μg/dL)** [5]				
HAPI	441	43.1±13.0	58%	39.2±19.9
**Urate (μmol/L)** [6]				
GUGC	110,347	37.7-76.4	0-75.6%	3.86-6.1

ATBC: Beta-Carotene Cancer Prevention Study; EPIC-Norfolk: the Norfolk arm of the European Prospective Investigation on Cancer study; EPIC: European Prospective Investigation into Cancer and Nutrition; PLCO: Prostate, Lung, Colorectal, and Ovarian Cancer Screening Trial; NHS: the Nurses' Health Study; HAPI: Heredity and Phenotype Intervention Heart Study; GUGC: the Global Urate Genetics Consortium

Table S2 The diagnostic information of five digestive system cancers in UK Biobank and FinnGen.

Outcome	UK Biobank				FinnGen					
	ICD-10	Sample Size	Cases	Controls	ICD-O-3			Sample Size	Cases	Controls
					ICD-8	ICD-9	ICD-10			
Esophagus cancer	C15	420,531	975	419,556	150	150	C15	174,238	232	174,006
Stomach cancer	C16	420,531	764	419,767	151	151	C16	174,639	633	174,006
Colon cancer	C18	420,531	3,759	416,772	153	153	C18	175,809	1,803	174,006
Pancreas cancer	C25	420,531	933	419,598	157	157	C25	174,611	605	174,006
Liver and intrahepatic bile ducts cancer	C22	420,531	539	419,992	155	155	C22	174,310	304	174,006

Table S3 The causal effect estimates of the associations between genetic instrumental variables for absolute circulating antioxidants and risk of five digestive system cancers.

Trait	SNP	Gene	Effect allele	F-statistic*	Exposure		Outcome									
							Esophageal cancer		Stomach cancer		Colon cancer		Pancreatic cancer		Liver cancer	
					Beta	SE	Beta	SE	Beta	SE	Beta	SE	Beta	SE	Beta	SE
**UK Biobank**																
α-tocopherol	rs11057830	SCARB1	A	16.00	0.040	0.010	-0.069	0.089	0.0002	0.075	0.002	0.034	0.102	0.067	0.045	0.089
α-tocopherol	rs2108622	CYP4F2	T	16.00	0.040	0.010	0.092	0.050	0.064	0.056	-0.021	0.026	0.088	0.051	0.026	0.066
α-tocopherol	rs964184	BUD13/ ZNF259/ APOA5	G	16.00	0.040	0.010	0.064	0.067	-0.015	0.076	0.035	0.035	-0.068	0.069	-0.133	0.090
Ascorbate	rs10051765	RGS14	C	31.04	0.039	0.007	-0.025	0.049	-0.072	0.055	0.032	0.025	0.026	0.049	-0.025	0.065
Ascorbate	rs10136000	AKT1	A	32.65	0.040	0.007	0.047	0.051	0.042	0.057	-0.046	0.026	-0.019	0.052	0.136	0.068
Ascorbate	rs117885456	SNRPF	A	42.25	0.078	0.012	-0.061	0.081	-0.067	0.091	-0.018	0.042	0.078	0.082	0.048	0.108
Ascorbate	rs13028225	SLC23A3	T	128.4	0.102	0.009	-0.089	0.066	-0.014	0.074	0.025	0.034	-0.015	0.067	0.035	0.088
Ascorbate	rs174547	FADS1	C	26.45	0.036	0.007	-0.076	0.048	-0.048	0.054	-0.039	0.025	0.117	0.049	0.017	0.064
Ascorbate	rs2559850	CHPT1	A	93.44	0.058	0.006	-0.089	0.047	-0.133	0.053	0.021	0.024	-0.061	0.048	-0.019	0.063
Ascorbate	rs33972313	SLC23A1	C	400.00	0.360	0.018	0.048	0.124	-0.109	0.14	-0.130	0.064	0.074	0.126	0.107	0.166
Ascorbate	rs56738967	MAF	C	34.31	0.041	0.007	-0.004	0.049	-0.056	0.055	-0.060	0.025	-0.029	0.050	0.155	0.066
Ascorbate	rs6693447	RER1	T	42.25	0.039	0.006	-0.027	0.046	0.028	0.052	-0.014	0.024	0.059	0.047	-0.091	0.061
Ascorbate	rs9895661	BCAS3	T	62.02	0.063	0.008	0.089	0.061	-0.053	0.069	-0.020	0.031	-0.032	0.062	-0.012	0.082
Retinol	rs10882272	RBP4	C	56.25	-0.030	0.004	0.081	0.047	0.131	0.053	-0.025	0.024	0.113	0.048	0.041	0.063
Retinol	rs1667255	TTR	C	56.25	0.030	0.004	0.075	0.047	0.084	0.053	-0.029	0.024	0.032	0.048	-0.033	0.063
Carotene	rs6564851	BCMO1	G	98.67	0.149	0.015	-0.013	0.046	0.037	0.055	-0.006	0.024	-0.028	0.047	-0.011	0.061
Carotene	rs7501331	BCMO1	T	17.69	-0.067	0.016	-0.013	0.054	-0.054	0.057	-0.001	0.028	0.072	0.055	0.034	0.072
Lycopene	rs2232315	G6PC2	A	24.34	0.740	0.150	-0.139	0.168	-0.070	0.188	0.091	0.086	-0.276	0.170	0.123	0.223
Lycopene	rs341075	-	A	26.19	-0.870	0.170	0.036	0.134	0.144	0.151	0.055	0.069	0.101	0.137	0.099	0.179
Lycopene	rs4635297	BC039545	A	27.04	0.260	0.050	-0.056	0.058	-0.021	0.066	0.021	0.03	-0.015	0.060	-0.058	0.078
Lycopene	rs6108801	-	C	28.44	-0.480	0.090	-0.021	0.123	0.107	0.138	0.045	0.063	0.131	0.125	0.127	0.165
Lycopene	rs7680948	SETD7	A	40.11	-0.190	0.030	0.001	0.051	0.043	0.058	-0.08	0.026	-0.058	0.052	0.031	0.069

Urate	rs10761587	-	C	46.42	-0.062	0.009	0.045	0.073	-0.017	0.082	0.112	0.038	-0.055	0.075	-0.04	0.098
Urate	rs11264341	TRIM46	T	66.19	-0.048	0.006	0.095	0.046	0.073	0.052	0.01	0.024	-0.067	0.047	-0.073	0.062
Urate	rs1165151	SLC17A1	G	290.26	0.092	0.005	-0.034	0.046	-0.012	0.052	-0.028	0.024	0.083	0.047	0.056	0.061
Urate	rs1171614	SLC16A9	C	108.63	0.074	0.007	-0.152	0.054	-0.028	0.061	-0.038	0.028	-0.012	0.055	-0.069	0.073
Urate	rs11722228	SLC2A9	T	1406.25	0.210	0.006	0.036	0.048	-0.011	0.054	0.025	0.025	0.046	0.049	0.029	0.064
Urate	rs1178977	BAZ1B	G	52.51	-0.050	0.007	0.062	0.057	-0.002	0.064	-0.006	0.029	0.007	0.058	-0.027	0.077
Urate	rs1260326	GCKR	C	196.00	-0.077	0.006	0.006	0.047	0.06	0.053	0.019	0.024	0.065	0.048	0.007	0.062
Urate	rs1394125	UBE2Q2	A	46.59	0.043	0.006	-0.044	0.048	-0.042	0.053	-0.002	0.024	5E-05	0.048	0.039	0.064
Urate	rs1471633	-	C	127.61	-0.061	0.005	0.017	0.046	-0.033	0.051	0.010	0.024	0.054	0.047	-0.110	0.061
Urate	rs17050272	LOC105373585	A	36.79	0.037	0.006	0.038	0.046	0.028	0.052	-0.014	0.024	-0.094	0.047	-0.075	0.062
Urate	rs17632159	LOC105379030	C	38.81	-0.038	0.006	-0.008	0.050	-0.028	0.056	-0.017	0.026	-0.032	0.051	-0.012	0.066
Urate	rs1825043	-	A	59.17	0.100	0.013	0.142	0.103	0.014	0.115	0.110	0.053	0.022	0.105	-0.144	0.137
Urate	rs2078267	SLC22A11	T	174.78	-0.078	0.006	0.016	0.046	0.067	0.052	-0.004	0.024	-0.003	0.047	-0.052	0.061
Urate	rs2231142	ABCG2	T	584.47	0.220	0.009	0.078	0.072	0.150	0.081	0.015	0.037	0.009	0.073	0.115	0.096
Urate	rs2307394	ORC4	C	37.70	0.035	0.006	-0.021	0.050	0.037	0.056	0.024	0.026	-0.007	0.051	-0.016	0.067
Urate	rs2941484	HNF4G	T	79.37	0.049	0.006	0.036	0.046	0.010	0.052	-0.03	0.024	0.031	0.047	-0.083	0.062
Urate	rs3741414	INHBC	T	102.88	-0.071	0.007	0.096	0.053	0.104	0.060	0.054	0.027	0.047	0.054	0.024	0.071
Urate	rs642803	OVOL1	T	63.41	-0.043	0.005	0.008	0.046	0.030	0.052	0.035	0.024	-0.022	0.047	-0.029	0.061
Urate	rs653178	ATXN2	T	44.44	-0.036	0.005	-0.020	0.046	0.009	0.051	0.076	0.023	-0.039	0.046	0.134	0.061
Urate	rs6598541	IGF1R	G	59.59	-0.044	0.006	0.012	0.048	-0.027	0.054	-0.011	0.025	-0.092	0.049	0.058	0.064
Urate	rs675209	-	C	103.25	-0.063	0.006	0.052	0.052	-0.008	0.058	-0.02	0.026	-0.053	0.052	0.013	0.069
Urate	rs6770152	-	T	73.47	-0.048	0.006	0.013	0.046	0.027	0.052	0.098	0.024	0.021	0.047	0.078	0.062
Urate	rs6830367	CLNK	C	50.17	0.051	0.007	0.014	0.060	0.009	0.068	0.02	0.031	-0.060	0.062	0.032	0.081
Urate	rs7193778	-	T	35.39	-0.047	0.008	-0.094	0.064	-0.111	0.072	-0.022	0.033	-0.088	0.065	-0.060	0.085
Urate	rs7224610	HLF	A	47.74	-0.038	0.006	-0.043	0.047	-0.050	0.053	0.015	0.024	-0.054	0.048	0.006	0.063
Urate	rs729761	POLR1C	G	53.31	0.046	0.006	-0.013	0.051	-0.015	0.057	0.034	0.026	-0.038	0.052	0.120	0.068
Urate	rs7654258	LOC107986260	T	98.01	-0.099	0.010	-0.060	0.088	-0.100	0.100	-0.016	0.046	0.157	0.090	-0.067	0.118
**FinnGen**																
α-Tocopherol	rs11057830	SCARB1	A	16.00	0.040	0.010	-0.082	0.137	0.090	0.284	-0.006	0.050	-0.049	0.085	0.042	0.119
α-Tocopherol	rs2108622	CYP4F2	T	16.00	0.040	0.010	0.033	0.117	0.041	0.071	-0.047	0.043	-0.004	0.073	0.104	0.102
α-Tocopherol	rs964184	BUD13/	G	16.00	0.040	0.010	0.081	0.132	0.127	0.080	-0.009	0.048	-0.025	0.082	-0.031	0.114

		ZNF259/ APOA5														
Ascorbate	rs10051765	RGS14	C	31.04	0.039	0.007	-0.074	0.096	0.114	0.058	-0.014	0.035	-0.042	0.060	-0.133	0.083
Ascorbate	rs10136000	AKT1	A	32.65	0.040	0.007	-0.007	0.103	0.162	0.063	-0.049	0.038	0.027	0.065	-0.147	0.09
Ascorbate	rs117885456	SNRPF	A	42.25	0.078	0.012	-0.221	0.169	-0.043	0.103	-0.078	0.061	-0.238	0.106	-0.145	0.148
Ascorbate	rs13028225	SLC23A3	T	128.4	0.102	0.009	0.137	0.130	0.020	0.100	0.037	0.047	0.024	0.081	-0.194	0.113
Ascorbate	rs174547	FADS1	C	26.45	0.036	0.007	0.103	0.095	0.074	0.058	-0.025	0.035	0.046	0.059	0.085	0.082
Ascorbate	rs2559850	CHPT1	A	93.44	0.058	0.006	0.012	0.095	-0.063	0.058	-0.016	0.035	0.038	0.059	-0.150	0.083
Ascorbate	rs33972313	SLC23A1	C	400.00	0.360	0.018	0.298	0.326	0.159	0.197	-0.158	0.118	-0.065	0.203	-0.163	0.283
Ascorbate	rs56738967	MAF	C	34.31	0.041	0.007	-0.042	0.101	0.017	0.062	-0.015	0.037	-0.043	0.063	-0.121	0.088
Ascorbate	rs6693447	RER1	T	42.25	0.039	0.006	0.107	0.094	0.011	0.057	0.026	0.034	0.022	0.059	0.119	0.082
Ascorbate	rs9895661	BCAS3	T	62.02	0.063	0.008	0.050	0.120	0.074	0.073	-0.036	0.044	0.015	0.075	0.020	0.104
Retinol	rs10882272	RBP4	C	56.25	-0.030	0.004	0.056	0.098	0.029	0.059	-0.026	0.036	0.116	0.061	0.030	0.085
Retinol	rs1667255	TTR	C	56.25	0.030	0.004	-0.148	0.155	-0.059	0.062	0.036	0.037	-0.093	0.064	-0.008	0.089
Carotene	rs6564851	BCMO1	G	98.67	0.149	0.015	-0.04	0.097	0.087	0.059	-0.058	0.035	0.074	0.06	0.136	0.084
Carotene	rs7501331	BCMO1	T	17.69	-0.067	0.016	0.252	0.101	0.064	0.061	0.043	0.037	-0.017	0.063	0.032	0.088
Lycopene	rs2232315	G6PC2	A	24.34	0.740	0.150	0.274	0.646	0.287	0.393	-0.347	0.235	0.006	0.421	0.595	0.577
Lycopene	rs341075	-	A	26.19	-0.870	0.170	-0.138	0.250	-0.002	0.150	0.061	0.09	0.364	0.155	-0.031	0.214
Lycopene	rs4635297	BC039545	A	27.04	0.260	0.050	-0.001	0.122	-0.179	0.074	-0.008	0.044	0.027	0.076	-0.195	0.107
Lycopene	rs6108801	-	C	28.44	-0.480	0.090	0.531	0.408	0.485	0.245	0.093	0.148	-0.182	0.254	0.358	0.363
Lycopene	rs7680948	SETD7	A	40.11	-0.190	0.030	0.064	0.109	-0.046	0.066	0.019	0.04	0.047	0.068	-0.068	0.096
Urate	rs10761587	-	C	46.42	-0.062	0.009	-0.107	0.134	-0.026	0.081	0.051	0.049	-0.108	0.083	-0.133	0.115
Urate	rs11264341	TRIM46	T	66.19	-0.048	0.006	-0.038	0.094	0.143	0.057	-0.054	0.034	0.003	0.058	-0.077	0.081
Urate	rs1165151	SLC17A1	G	290.26	0.092	0.005	-0.081	0.096	-0.048	0.058	0.006	0.035	-0.047	0.06	0.070	0.084
Urate	rs1171614	SLC16A9	C	108.63	0.074	0.007	-0.001	0.129	0.037	0.078	-0.038	0.047	-0.008	0.08	0.225	0.112
Urate	rs11722228	SLC2A9	T	1406.25	0.210	0.006	-0.290	0.100	-0.030	0.060	-0.056	0.036	0.069	0.062	0.016	0.087
Urate	rs1178977	BAZ1B	G	52.51	-0.050	0.007	0.142	0.122	0.078	0.074	-0.009	0.045	0.004	0.077	-0.114	0.107
Urate	rs1260326	GCKR	C	196.00	-0.077	0.006	0.016	0.098	-0.005	0.060	0.105	0.036	0.018	0.061	0.022	0.085
Urate	rs1394125	UBE2Q2	A	46.59	0.043	0.006	-0.15	0.107	0.083	0.065	0.019	0.039	0.005	0.066	-0.035	0.093
Urate	rs1471633	-	C	127.61	-0.061	0.005	0.072	0.098	0.002	0.059	0.019	0.036	-0.065	0.061	0.125	0.086
Urate	rs17050272	LOC105373585	A	36.79	0.037	0.006	0.076	0.094	0.019	0.057	-0.041	0.034	0.059	0.059	0.104	0.082

Urate	rs17632159	LOC105379030	C	38.81	-0.038	0.006	0.012	0.108	-0.036	0.065	-0.006	0.039	0.007	0.067	0.034	0.094
Urate	rs1825043	-	A	59.17	0.100	0.013	-0.349	0.176	0.062	0.107	0.037	0.065	0.083	0.109	0.065	0.153
Urate	rs2078267	SLC22A11	T	174.78	-0.078	0.006	-0.037	0.095	-0.043	0.057	0.030	0.035	-0.003	0.059	0.055	0.083
Urate	rs2231142	ABCG2	T	584.47	0.220	0.009	-0.205	0.176	-0.02	0.107	-0.043	0.064	0.214	0.110	0.387	0.155
Urate	rs2307394	ORC4	C	37.70	0.035	0.006	-0.022	0.099	-0.035	0.060	-0.054	0.036	0.039	0.062	-0.117	0.086
Urate	rs2941484	HNF4G	T	79.37	0.049	0.006	-0.065	0.094	0.025	0.057	0.016	0.034	-0.002	0.058	-0.061	0.082
Urate	rs3741414	INHBC	T	102.88	-0.071	0.007	-0.100	0.110	0.070	0.067	0.039	0.040	0.027	0.069	-0.008	0.096
Urate	rs642803	OVOL1	T	63.41	-0.043	0.005	-0.078	0.096	-0.014	0.058	0.010	0.035	0.025	0.06	-0.076	0.084
Urate	rs653178	ATXN2	T	44.44	-0.036	0.005	0.092	0.095	-0.023	0.058	0.029	0.035	0.052	0.059	0.007	0.083
Urate	rs6598541	IGF1R	G	59.59	-0.044	0.006	0.010	0.098	-0.110	0.060	-0.023	0.036	-0.042	0.061	0.038	0.085
Urate	rs675209	-	C	103.25	-0.063	0.006	-0.097	0.101	-0.104	0.061	-0.013	0.037	-0.063	0.062	-0.005	0.087
Urate	rs6770152	-	T	73.47	-0.048	0.006	0.038	0.096	-0.011	0.058	0.009	0.035	0.040	0.060	-0.121	0.084
Urate	rs6830367	CLNK	C	50.17	0.051	0.007	0.161	0.117	-0.027	0.071	0.012	0.043	-0.111	0.073	0.114	0.102
Urate	rs7193778	-	T	35.39	-0.047	0.008	-0.144	0.136	-0.042	0.083	-0.125	0.05	-0.048	0.085	0.283	0.119
Urate	rs7224610	HLF	A	47.74	-0.038	0.006	-0.043	0.095	-0.049	0.058	0.043	0.035	0.060	0.059	0.012	0.083
Urate	rs729761	POLR1C	G	53.31	0.046	0.006	-0.045	0.103	-0.023	0.063	0.020	0.038	0.060	0.064	0.002	0.090
Urate	rs7654258	LOC107986260	T	98.01	-0.099	0.010	0.130	0.167	-0.069	0.102	-0.122	0.061	0.062	0.104	-0.060	0.146

*: The F-statistic for each SNPs was calculated by the following formula: F-statistic = $Beta^2/SE^2$

Table S4 The causal effect estimates of the associations between genetic instrumental variables for circulating antioxidant metabolites and risk of five digestive system cancers.

Trait	SNP	Gene	Effect allele	F-statistic *	Exposure		Outcome									
							Esophageal cancer		Stomach cancer		Colon cancer		Pancreatic cancer		Liver cancer	
					Beta	SE	Beta	SE	Beta	SE	Beta	SE	Beta	SE	Beta	SE
**UK Biobank**																
α-Tocopherol	rs10163969	-	T	19.36	-0.035	0.008	-0.176	0.119	0.031	0.134	0.020	0.061	0.115	0.121	0.201	0.159
α-Tocopherol	rs10245705	LOC105375199	T	27.25	-0.066	0.013	-0.119	0.169	-0.204	0.191	0.012	0.087	0.132	0.172	0.248	0.226
α-Tocopherol	rs10935814	-	A	19.87	-0.037	0.008	0.010	0.070	0.019	0.079	0.029	0.036	0.185	0.071	0.109	0.094
α-Tocopherol	rs11145330	-	C	22.7	-0.032	0.007	0.018	0.072	0.010	0.081	-0.044	0.037	0.028	0.073	0.078	0.096
α-Tocopherol	rs11992435	-	G	20.63	-0.033	0.007	0.160	0.099	-0.011	0.111	-0.044	0.051	0.093	0.101	-0.003	0.133
α-Tocopherol	rs1404410	-	G	20.6	0.024	0.005	0.025	0.057	0.029	0.065	0.031	0.030	0.030	0.059	-0.049	0.077
α-Tocopherol	rs1532701	SLC6A2	A	20.85	0.014	0.003	-0.019	0.046	-0.056	0.052	-0.012	0.024	0.042	0.047	0.039	0.061
α-Tocopherol	rs2074731	SF3A1	A	22.26	-0.018	0.004	-0.006	0.062	-0.016	0.070	-0.009	0.032	-0.068	0.063	0.058	0.083
α-Tocopherol	rs261342	LIPC/ LIPC-AS1	C	20.86	-0.017	0.004	-0.078	0.055	-0.015	0.062	0.006	0.028	0.003	0.056	-0.012	0.074
α-Tocopherol	rs7238006	-	C	24.3	-0.028	0.006	-0.041	0.091	-0.196	0.102	-0.021	0.046	-0.113	0.092	-0.126	0.121
α-Tocopherol	rs7930821	ZDHHC13	T	19.98	0.067	0.015	-0.294	0.166	0.062	0.187	-0.135	0.086	-0.137	0.169	-0.149	0.222
γ-Tocopherol	rs10077932	-	T	21.35	-0.040	0.009	0.064	0.059	0.0001	0.067	0.063	0.030	-0.106	0.06	0.029	0.079
γ-Tocopherol	rs1013104	-	T	21.16	-0.021	0.005	-0.017	0.046	0.040	0.052	0.007	0.024	-0.099	0.047	-0.008	0.062
γ-Tocopherol	rs10466757	BCAT1	T	19.66	-0.063	0.014	0.068	0.053	0.132	0.059	-0.037	0.027	0.045	0.053	-0.019	0.070
γ-Tocopherol	rs10492212	-	T	19.53	-0.027	0.006	0.058	0.062	0.065	0.070	-0.036	0.032	-0.039	0.064	-0.060	0.083
γ-Tocopherol	rs10520845	MYO10	A	20.78	0.191	0.042	-0.166	0.213	-0.151	0.240	0.027	0.110	-0.024	0.217	0.214	0.286
γ-Tocopherol	rs1060467	CYP4F11	G	26.81	-0.023	0.005	-0.046	0.046	-0.017	0.052	0.026	0.024	-0.089	0.047	-0.066	0.062
γ-Tocopherol	rs13336771	LONP2	A	20.15	0.062	0.014	0.008	0.064	-0.003	0.072	0.006	0.033	0.042	0.065	-0.118	0.085
γ-Tocopherol	rs261301	-	C	22.56	-0.032	0.007	-0.029	0.066	0.033	0.074	-0.018	0.034	0.195	0.067	-0.069	0.088
γ-Tocopherol	rs2794327	MEGF6	T	19.69	-0.036	0.008	0.003	0.049	-0.017	0.076	0.005	0.025	0.085	0.05	0.032	0.066
γ-Tocopherol	rs5994305	SEC14L2	G	24.52	-0.031	0.006	-0.018	0.061	-0.048	0.069	-0.018	0.031	-0.077	0.062	0.034	0.082
γ-Tocopherol	rs6821770	SORCS2	A	19.52	0.038	0.009	0.015	0.060	-0.089	0.067	-0.036	0.031	-0.008	0.061	-0.001	0.080

γ-Tocopherol	rs7038957	-	C	21.43	0.029	0.006	0.002	0.061	0.082	0.069	0.045	0.031	0.013	0.062	0.016	0.082
γ-Tocopherol	rs7350776	RYR3	G	21.12	-0.024	0.005	-0.015	0.050	0.042	0.056	0.026	0.026	0.010	0.051	-0.036	0.066
Ascorbate	rs11167905	-	C	24.03	-0.080	0.016	0.062	0.063	-0.043	0.071	0.055	0.033	-0.083	0.064	0.007	0.084
Ascorbate	rs13069990	LSAMP	T	21.16	-0.051	0.011	0.073	0.047	0.036	0.053	0.052	0.024	0.047	0.048	-0.098	0.063
Ascorbate	rs13103690	SLC2A9	G	20.77	0.047	0.010	0.045	0.046	0.008	0.051	-0.015	0.024	-0.059	0.047	-0.091	0.061
Ascorbate	rs2070006	FGA	C	20.9	-0.051	0.011	0.014	0.047	-0.024	0.053	-0.027	0.024	-0.076	0.048	0.070	0.063
Ascorbate	rs577596	-	A	24.74	-0.057	0.011	0.005	0.048	0.020	0.054	0.011	0.025	-0.046	0.049	-0.055	0.064
Ascorbate	rs6713914	LOC105374754	C	26.13	-0.059	0.012	0.014	0.046	0.012	0.051	0.018	0.024	-0.036	0.047	-0.011	0.061
Ascorbate	rs6826474	-	T	23.06	-0.138	0.029	-0.084	0.143	0.054	0.161	0.014	0.074	0.023	0.145	0.204	0.191
Ascorbate	rs6834631	LOC107986309	G	23.93	-0.131	0.027	0.210	0.110	-0.210	0.124	-0.033	0.057	-0.118	0.113	-0.027	0.148
Ascorbate	rs7112460	HYLS1	T	23.63	0.108	0.022	-0.112	0.094	-0.092	0.106	-0.007	0.048	-0.032	0.096	-0.013	0.126
Ascorbate	rs8057559	ZFHX3	T	19.74	0.140	0.031	-0.317	0.140	-0.139	0.157	-0.075	0.072	0.076	0.143	0.422	0.187
Ascorbate	rs808686	-	A	21.83	0.060	0.013	-0.028	0.046	-0.034	0.051	0.020	0.024	-0.004	0.047	-0.002	0.061
Ascorbate	rs8105491	-	T	22.24	-0.070	0.015	0.044	0.064	-0.041	0.072	0.014	0.033	0.012	0.065	0.011	0.085
Ascorbate	rs9419004	ADGRA1	C	20.35	-0.254	0.056	-0.061	0.051	0.041	0.057	0.002	0.026	-0.067	0.052	0.021	0.068
Ascorbate	rs9606290	LOC105372863	A	20.4	0.159	0.035	-0.030	0.053	0.007	0.059	-0.012	0.027	0.104	0.054	-0.006	0.071
Retinol	rs10019071	-	A	16.72	0.657	0.161	-0.198	0.167	0.126	0.187	-0.032	0.085	0.085	0.169	-0.330	0.223
Retinol	rs112293959	ZFAND6	G	10.88	-0.429	0.130	-0.065	0.129	-0.116	0.145	0.013	0.066	-0.010	0.131	0.238	0.172
Retinol	rs114515641	IGSF11	G	10.32	0.414	0.129	-0.128	0.134	-0.273	0.151	0.117	0.069	-0.152	0.136	-0.221	0.178
Retinol	rs1153379	-	A	14.97	-0.323	0.083	-0.010	0.095	-0.005	0.107	0.076	0.049	0.051	0.097	-0.205	0.127
Retinol	rs117468033	-	T	26.23	-0.961	0.188	-	-	-	-	-	-	-	-	-	-
Retinol	rs1176744	HTR3B	C	20.97	-0.207	0.045	0.023	0.049	0.010	0.055	-0.013	0.025	0.046	0.05	-0.022	0.066
Retinol	rs118025446	CEP162	A	17.38	-0.481	0.115	0.079	0.116	0.165	0.131	0.033	0.06	-0.033	0.119	0.221	0.156
Retinol	rs12955464	-	G	14.79	-0.234	0.061	-0.012	0.065	0.077	0.073	-0.021	0.033	0.067	0.066	-0.012	0.087
Retinol	rs139726207	-	G	11.21	0.370	0.110	-0.181	0.095	-0.067	0.107	0.042	0.049	0.103	0.096	0.047	0.127
Retinol	rs149113848	-	G	13.21	-0.964	0.265	-0.468	0.268	-0.004	0.300	-0.031	0.137	0.135	0.271	0.258	0.356
Retinol	rs149478645	CTTNBP2	G	12.79	-0.507	0.142	0.024	0.154	-0.135	0.173	0.072	0.079	0.121	0.157	-0.169	0.206
Retinol	rs17005512	-	C	13.33	-0.218	0.060	-0.064	0.063	-0.015	0.071	0.022	0.033	-0.011	0.064	-0.009	0.085

Retinol	rs1842947	OR3A3	G	20.06	-0.192	0.043	-0.015	0.046	-0.004	0.052	-0.045	0.024	0.027	0.047	0.088	0.061
Retinol	rs2147337	SIRPG	G	12.68	0.158	0.044	-0.054	0.048	-0.063	0.053	-0.0003	0.024	0.032	0.048	0.101	0.064
Retinol	rs2367816	LOC105374455	G	19.64	0.228	0.051	-0.066	0.054	0.018	0.060	0.019	0.028	0.009	0.055	0.191	0.072
Retinol	rs2417325	-	T	15.49	0.329	0.084	-0.113	0.089	0.007	0.101	-0.034	0.046	0.002	0.091	0.037	0.119
Retinol	rs3890033	TNK2	C	11.76	0.144	0.042	-0.030	0.046	-0.094	0.052	-0.026	0.024	-0.048	0.047	0.045	0.062
Retinol	rs3898702	LOC101927394	T	16.35	-0.217	0.054	0.082	0.057	0.043	0.064	-0.024	0.029	0.039	0.058	-0.011	0.076
Retinol	rs4135385	CTNNB1	G	18.75	0.212	0.049	0.040	0.054	0.012	0.061	0.077	0.028	-0.012	0.055	0.065	0.072
Retinol	rs568632536	-	T	14.35	0.531	0.140	-	-	-	-	-	-	-	-	-	-
Retinol	rs58411567	DNAH10	A	15.92	-0.208	0.052	0.051	0.054	0.047	0.061	0.039	0.028	0.041	0.056	-0.060	0.073
Retinol	rs6550239	RAB5A	A	14.51	-0.183	0.048	0.018	0.055	-0.006	0.062	0.044	0.028	0.078	0.056	-0.038	0.073
Retinol	rs75308833	-	T	11.27	-0.494	0.147	-0.041	0.144	0.021	0.162	-0.066	0.074	-0.170	0.146	-0.303	0.193
Retinol	rs7926028	-	T	10.39	-0.135	0.042	-0.042	0.046	-0.085	0.051	-0.003	0.023	0.013	0.046	-0.026	0.061
Retinol	rs945817	LOC105377858	A	25.32	-0.275	0.055	0.130	0.058	0.218	0.065	-0.006	0.03	-0.041	0.059	0.085	0.077
Retinol	rs9586119	-	C	18.24	0.355	0.083	-0.065	0.081	0.062	0.091	-0.048	0.042	-0.150	0.083	0.059	0.109
Urate	rs10490734	THSD7B	G	20.52	-0.008	0.002	-0.098	0.061	-0.125	0.069	0.033	0.032	0.046	0.062	0.039	0.082
Urate	rs10844317	DNM1L	T	22.83	-0.009	0.002	0.046	0.066	0.173	0.074	-0.003	0.034	0.046	0.067	-0.159	0.088
Urate	rs11130013	-	T	20.25	-0.008	0.002	0.051	0.063	0.089	0.071	-0.022	0.032	-0.002	0.064	0.092	0.084
Urate	rs11741189	RASGEF1C	T	26.19	-0.009	0.002	0.079	0.061	0.077	0.069	0.001	0.032	0.022	0.062	-0.028	0.082
Urate	rs1286087	RPS6KA5	C	20.52	-0.008	0.002	0.007	0.064	0.075	0.072	0.002	0.033	-0.051	0.065	-0.124	0.085
Urate	rs13318012	-	C	21.05	0.008	0.002	-0.040	0.058	-0.013	0.065	-0.024	0.030	0.001	0.059	-0.046	0.078
Urate	rs16953268	LINC02169	A	19.56	-0.012	0.003	-0.105	0.091	-0.219	0.102	0.032	0.047	-0.051	0.092	0.170	0.121
Urate	rs2231142	ABCG2	T	24.59	0.012	0.002	0.078	0.072	0.150	0.081	0.015	0.037	0.009	0.073	0.115	0.096
Urate	rs233340	-	A	23.11	-0.013	0.003	-0.072	0.088	-0.101	0.099	-0.029	0.045	0.036	0.09	0.057	0.118
Urate	rs235320	PTTG1IP	G	21.6	0.008	0.002	-0.044	0.058	0.030	0.065	-0.029	0.03	0.040	0.059	0.003	0.077
Urate	rs510032	LOC105373116	C	20.25	-0.009	0.002	0.022	0.058	0.066	0.066	0.028	0.03	-0.057	0.059	-0.018	0.078
Urate	rs6731691	ITGA6	A	19.75	-0.008	0.002	-0.014	0.061	-0.053	0.069	-0.002	0.032	0.003	0.063	0.021	0.082

Urate	rs6927639	-	G	18.78	0.008	0.002	-0.059	0.064	-0.072	0.072	-0.043	0.033	0.020	0.065	0.172	0.085
Urate	rs7906282	LOC105376440	T	25	0.008	0.002	-0.002	0.049	0.009	0.055	-0.007	0.025	-0.015	0.05	-0.081	0.066
Urate	rs872114	-	C	22.35	0.021	0.004	0.177	0.135	0.136	0.152	0.017	0.070	0.070	0.138	0.289	0.18
Urate	rs938554	SLC2A9	G	419.04	0.035	0.002	0.041	0.055	0.009	0.062	-0.007	0.029	0.010	0.056	0.055	0.074
Urate	rs9549021	-	C	21.67	0.014	0.003	0.084	0.047	-0.053	0.053	-0.018	0.024	0.018	0.048	-0.036	0.063
Urate	rs9557545	NALCN-AS1	A	20.25	0.018	0.004	0.183	0.137	0.144	0.154	-0.062	0.07	-0.187	0.139	-0.302	0.183
**FinnGen**																
α-Tocopherol	rs10163969	-	T	19.36	-0.035	0.008	0.156	0.144	-0.137	0.088	-0.011	0.053	0.089	0.089	-0.183	0.124
α-Tocopherol	rs10245705	LOC105375199	T	27.25	-0.066	0.013	-0.307	0.388	0.152	0.233	-0.143	0.139	-0.627	0.252	-7E-04	0.359
α-Tocopherol	rs10935814	-	A	19.87	-0.037	0.008	-0.238	0.148	0.061	0.09	-0.110	0.054	0.040	0.092	0.006	0.129
α-Tocopherol	rs11145330	-	C	22.7	-0.032	0.007	-0.232	0.131	-0.087	0.079	0.075	0.048	-0.094	0.081	0.073	0.113
α-Tocopherol	rs11992435	-	G	20.63	-0.033	0.007	-0.166	0.225	-0.030	0.138	-0.179	0.083	-0.133	0.141	0.171	0.197
α-Tocopherol	rs1404410	-	G	20.6	0.024	0.005	-0.075	0.117	-0.100	0.071	-0.005	0.043	0.130	0.073	-0.058	0.102
α-Tocopherol	rs1532701	SLC6A2	A	20.85	0.014	0.003	-0.034	0.094	0.008	0.057	0.053	0.034	-0.069	0.059	0.141	0.082
α-Tocopherol	rs2074731	SF3A1	A	22.26	-0.018	0.004	0.137	0.121	0.016	0.073	0.012	0.044	-0.046	0.075	0.019	0.104
α-Tocopherol	rs261342	LIPC/	C	20.86	-0.017	0.004	-0.005	0.113	-0.063	0.069	0.017	0.041	0.054	0.07	-0.034	0.098
α-Tocopherol	rs7238006	LIPC-AS1	C	24.3	-0.028	0.006	0.048	0.136	-4E-04	0.083	-0.004	0.05	-0.065	0.085	0.082	0.119
α-Tocopherol	rs7930821	-	T	19.98	0.067	0.015	0.762	0.447	0.018	0.275	0.054	0.165	-0.295	0.288	-0.346	0.411
γ-Tocopherol	rs10077932	ZDHHC13	T	21.35	-0.040	0.009	-0.054	0.12	-0.083	0.073	-0.044	0.044	-0.063	0.075	0.004	0.105
γ-Tocopherol	rs1013104	-	T	21.16	-0.021	0.005	0.071	0.094	0.029	0.057	0.025	0.034	-0.015	0.059	0.098	0.082
γ-Tocopherol	rs10466757	-	T	19.66	-0.063	0.014	-0.147	0.106	0.028	0.065	0.011	0.039	-0.011	0.066	-0.172	0.093
γ-Tocopherol	rs10492212	BCAT1	T	19.53	-0.027	0.006	0.124	0.12	0.16	0.073	0.040	0.044	0.019	0.075	0.043	0.104
γ-Tocopherol	rs10520845	-	A	20.78	0.191	0.042	0.236	0.362	0.01	0.222	-0.047	0.134	0.201	0.224	-0.112	0.316
γ-Tocopherol	rs1060467	MYO10	G	26.81	-0.023	0.005	0.039	0.094	0.062	0.057	0.002	0.034	0.097	0.059	-0.104	0.082
γ-Tocopherol	rs13336771	CYP4F11	A	20.15	0.062	0.014	0.083	0.126	0.024	0.077	0.098	0.046	-0.028	0.078	-0.027	0.11
γ-Tocopherol	rs261301	LONP2	C	22.56	-0.032	0.007	-0.052	0.125	0.009	0.076	0.004	0.046	0.036	0.078	-0.117	0.109
γ-Tocopherol	rs2794327	-	T	19.69	-0.036	0.008	-0.057	0.100	0.06	0.061	0.010	0.037	0.003	0.062	0.034	0.087
γ-Tocopherol	rs5994305	MEGF6	G	24.52	-0.031	0.006	0.137	0.120	0.038	0.073	-0.012	0.044	-0.048	0.074	0.031	0.103
γ-Tocopherol	rs6821770	SEC14L2	A	19.52	0.038	0.009	0.237	0.112	0.071	0.068	-0.005	0.041	0.010	0.070	-0.029	0.097

γ-Tocopherol	rs7038957	SORCS2	C	21.43	0.029	0.006	-0.129	0.130	0.033	0.079	0.045	0.047	-0.062	0.081	-0.134	0.112
γ-Tocopherol	rs7350776	-	G	21.12	-0.024	0.005	0.080	0.099	0.11	0.06	0.011	0.036	-0.017	0.062	-0.072	0.087
Ascorbate	rs11167905	RYR3	C	24.03	-0.080	0.016	0.197	0.141	0.031	0.086	-0.015	0.051	0.011	0.088	-0.009	0.123
Ascorbate	rs13069990	-	T	21.16	-0.051	0.011	0.084	0.097	0.039	0.059	0.045	0.035	-0.052	0.06	0.088	0.084
Ascorbate	rs13103690	LSAMP	G	20.77	0.047	0.010	0.076	0.094	0.064	0.057	0.043	0.034	-0.011	0.059	0.012	0.082
Ascorbate	rs2070006	SLC2A9	C	20.9	-0.051	0.011	-0.055	0.094	0.007	0.057	0.017	0.034	0.071	0.058	-0.047	0.082
Ascorbate	rs577596	FGA	A	24.74	-0.057	0.011	-0.151	0.096	-0.062	0.058	-0.013	0.035	-0.013	0.06	0.006	0.083
Ascorbate	rs6713914	-	C	26.13	-0.059	0.012	0.236	0.095	0.004	0.057	-0.005	0.035	0.03	0.059	-0.007	0.082
Ascorbate	rs6826474	LOC105374754	T	23.06	-0.138	0.029	0.612	0.331	0.252	0.201	-0.122	0.119	0.291	0.208	0.101	0.295
Ascorbate	rs6834631	-	G	23.93	-0.131	0.027	0.195	0.335	-0.114	0.201	0.085	0.122	-0.326	0.213	-0.219	0.299
Ascorbate	rs7112460	LOC107986309	T	23.63	0.108	0.022	-0.008	0.219	-0.115	0.132	-0.098	0.079	0.107	0.137	-0.072	0.191
Ascorbate	rs8057559	HYLS1	T	19.74	0.140	0.031	-0.621	0.291	-0.158	0.176	-0.128	0.106	0.039	0.179	-0.091	0.250
Ascorbate	rs808686	ZFHX3	A	21.83	0.060	0.013	0.027	0.096	-0.020	0.058	0.003	0.035	0.003	0.060	-0.111	0.084
Ascorbate	rs8105491	-	T	22.24	-0.070	0.015	-0.176	0.123	0.123	0.074	0.116	0.045	-0.075	0.077	-0.049	0.107
Ascorbate	rs9419004	-	C	20.35	-0.254	0.056	-0.014	0.111	0.076	0.067	-0.01	0.041	0.065	0.069	0.137	0.097
Ascorbate	rs9606290	ADGRA1	A	20.4	0.159	0.035	0.034	0.128	-0.007	0.078	-0.013	0.046	-0.039	0.080	-0.068	0.112
Retinol	rs10019071	-	A	16.72	0.657	0.161	-0.005	0.586	0.441	0.351	-0.389	0.213	-0.015	0.384	-0.262	0.541
Retinol	rs112293959	ZFAND6	G	10.88	-0.429	0.130	0.266	0.318	-0.089	0.196	-0.018	0.115	-0.155	0.201	0.342	0.28
Retinol	rs114515641	IGSF11	G	10.32	0.414	0.129	0.156	0.285	0.455	0.172	0.062	0.103	-0.108	0.181	-0.299	0.251
Retinol	rs1153379	-	A	14.97	-0.323	0.083	0.118	0.203	0.132	0.123	-0.039	0.074	0.11	0.125	-0.141	0.174
Retinol	rs117468033	-	T	26.23	-0.961	0.188	-0.118	0.413	0.123	0.247	-0.143	0.149	-0.002	0.254	-0.262	0.352
Retinol	rs1176744	HTR3B	C	20.97	-0.207	0.045	-0.044	0.102	-0.011	0.062	-0.007	0.037	-0.048	0.064	0.145	0.089
Retinol	rs118025446	CEP162	A	17.38	-0.481	0.115	-0.183	0.327	0.157	0.198	-0.085	0.119	-0.413	0.206	-0.094	0.292
Retinol	rs12955464	-	G	14.79	-0.234	0.061	0.023	0.141	0.014	0.086	-0.067	0.051	0.06	0.088	-0.031	0.124
Retinol	rs139726207	-	G	11.21	0.370	0.110	0.102	0.176	-0.015	0.106	-0.045	0.064	-0.028	0.109	0.127	0.154
Retinol	rs149113848	-	G	13.21	-0.964	0.265	-1.034	0.770	0.281	0.467	-0.289	0.276	0.177	0.482	-0.552	0.680
Retinol	rs149478645	CTTNBP2	G	12.79	-0.507	0.142	0.356	0.416	-0.069	0.248	0.306	0.148	-0.27	0.263	0.743	0.366
Retinol	rs17005512	-	C	13.33	-0.218	0.060	0.080	0.122	0.079	0.074	0.041	0.044	-0.077	0.076	0.109	0.106
Retinol	rs1842947	OR3A3	G	20.06	-0.192	0.043	0.101	0.095	-0.029	0.058	0.007	0.035	0.025	0.059	0.049	0.083

Retinol	rs2147337	SIRPG	G	12.68	0.158	0.044	-0.049	0.102	0.027	0.062	0.009	0.037	0.06	0.064	-0.038	0.089
Retinol	rs2367816	LOC105374455	G	19.64	0.228	0.051	-0.109	0.110	0.048	0.067	-0.025	0.040	0.057	0.069	0.031	0.096
Retinol	rs2417325	-	T	15.49	0.329	0.084	0.298	0.158	0.087	0.096	0.064	0.058	-0.033	0.098	0.004	0.137
Retinol	rs3890033	TNK2	C	11.76	0.144	0.042	0.024	0.094	0.076	0.057	-0.020	0.035	0.032	0.059	0.013	0.082
Retinol	rs3898702	LOC101927394	T	16.35	-0.217	0.054	-0.089	0.112	0.002	0.068	0.012	0.041	-0.029	0.070	-0.107	0.097
Retinol	rs4135385	CTNNB1	G	18.75	0.212	0.049	0.028	0.116	-0.038	0.070	0.020	0.042	-0.081	0.073	-0.092	0.101
Retinol	rs568632536	-	T	14.35	0.531	0.140	-	-	-	-	-	-	-	-	-	-
Retinol	rs58411567	DNAH10	A	15.92	-0.208	0.052	0.074	0.110	-0.034	0.067	-0.010	0.04	0.097	0.069	0.182	0.096
Retinol	rs6550239	RAB5A	A	14.51	-0.183	0.048	0.154	0.107	-0.019	0.065	0.007	0.039	-0.031	0.066	0.153	0.093
Retinol	rs75308833	-	T	11.27	-0.494	0.147	-0.643	0.388	0.056	0.238	0.141	0.141	-0.283	0.241	0.265	0.342
Retinol	rs7926028	-	T	10.39	-0.135	0.042	-0.155	0.225	0.191	0.137	0.094	0.082	-0.134	0.141	0.137	0.197
Retinol	rs945817	LOC105377858	A	25.32	-0.275	0.055	0.039	0.148	-0.106	0.09	-0.122	0.054	-0.147	0.093	0.203	0.131
Retinol	rs9586119	-	C	18.24	0.355	0.083	0.178	0.148	0.084	0.09	-0.086	0.054	-0.052	0.092	0.099	0.128
Urate	rs10490734	THSD7B	G	20.52	-0.008	0.002	0.113	0.114	-0.028	0.069	0.075	0.041	0.080	0.070	-0.146	0.098
Urate	rs10844317	DNM1L	T	22.83	-0.009	0.002	-0.184	0.150	0.141	0.091	0.032	0.054	0.098	0.094	0.161	0.131
Urate	rs11130013	-	T	20.25	-0.008	0.002	0.040	0.161	-0.045	0.098	0.057	0.058	0.055	0.101	-0.064	0.141
Urate	rs11741189	RASGEF1C	T	26.19	-0.009	0.002	-0.171	0.133	-0.090	0.08	-0.002	0.048	-0.011	0.083	0.171	0.116
Urate	rs1286087	RPS6KA5	C	20.52	-0.008	0.002	-0.274	0.159	0.091	0.097	-0.059	0.058	0.066	0.099	-0.161	0.139
Urate	rs13318012	-	C	21.05	0.008	0.002	0.119	0.125	0.019	0.076	-0.022	0.045	-0.026	0.078	0.165	0.108
Urate	rs16953268	LINC02169	A	19.56	-0.012	0.003	0.238	0.221	0.004	0.135	-0.149	0.08	0.055	0.140	-0.092	0.196
Urate	rs2231142	ABCG2	T	24.59	0.012	0.002	-0.205	0.176	-0.020	0.107	-0.043	0.064	0.214	0.110	0.387	0.155
Urate	rs233340	-	A	23.11	-0.013	0.003	0.209	0.145	-0.082	0.088	0.077	0.053	0.079	0.090	-0.039	0.126
Urate	rs235320	PTTG1IP	G	21.6	0.008	0.002	0.047	0.143	-0.019	0.088	-0.04	0.052	-0.119	0.090	0.078	0.126
Urate	rs510032	LOC105373116	C	20.25	-0.009	0.002	6E-04	0.115	-0.016	0.07	-0.028	0.042	-0.032	0.071	0.014	0.099
Urate	rs6731691	ITGA6	A	19.75	-0.008	0.002	0.154	0.130	-0.056	0.079	0.082	0.047	-0.097	0.082	-0.066	0.114
Urate	rs6927639	-	G	18.78	0.008	0.002	-0.070	0.107	0.121	0.065	0.002	0.039	0.041	0.067	0.107	0.093

Urate	rs7906282	LOC105376440	T	25	0.008	0.002	-0.037	0.111	-0.099	0.067	-0.052	0.04	-0.092	0.069	0.034	0.096
Urate	rs872114	-	C	22.35	0.021	0.004	-0.282	0.508	0.175	0.305	-0.007	0.181	-0.174	0.324	-0.633	0.454
Urate	rs938554	SLC2A9	G	419.04	0.035	0.002	-0.052	0.124	0.069	0.075	0.057	0.045	0.120	0.077	-0.108	0.108
Urate	rs9549021	-	C	21.67	0.014	0.003	0.133	0.097	0.058	0.059	0.007	0.035	0.054	0.060	0.001	0.084
Urate	rs9557545	NALCN-AS1	A	20.25	0.018	0.004	-0.138	0.139	-0.027	0.084	0.077	0.051	-0.031	0.085	0.244	0.119

*: The F-statistic for each SNPs was calculated by the following formula: F-statistic = $Beta^2/SE^2$

Table S5 The complementary MR analyses results of the causal effects of absolute circulating antioxidants on five digestive system cancers.

MR methods	α-Tocopherol (3 SNPs)		Ascorbate (10 SNPs)		Retinol (2 SNPs)		β-Carotene (2 SNPs)		Lycopene (5 SNPs)		Urate (27 SNPs)	
	OR (95%CI)	*p*-Value	OR (95%CI)	*p*-Value	OR (95%CI)	*p*-Value	OR (95%CI)	*p*-Value	OR (95%CI)	*p*-Value	OR (95%CI)	*p*-Value
**UK Biobank**												
**Esophageal cancer**												
Maximum likelihood	1.155 (0.964,1.385)	0.118	0.797 (0.497,1.276)	0.344	0.991 (0.793,1.238)	0.936	0.995 (0.941,1.052)	0.86	0.921 (0.763,1.111)	0.39	0.946 (0.733,1.221)	0.672
MR-Egger	-	-	1.098 (0.482,2.503)	0.829	-	-	-	-	0.933 (0.661,1.319)	0.722	1.260 (0.780,2.036)	0.354
Weighted median	1.206 (0.953,1.526)	0.119	0.991 (0.539,1.824)	0.977	-	-	-	-	0.939 (0.744,1.184)	0.592	1.088 (0.751,1.577)	0.655
Penalised weighted median	1.206 (0.972,1.496)	0.088	0.991 (0.548,1.794)	0.977	-	-	-	-	0.939 (0.751,1.174)	0.579	1.112 (0.761,1.625)	0.584
MR-PRESSO	-	-	-	-	-	-	-	-	-	-	-	-
**Stomach cancer**												
Maximum likelihood	1.071 (0.886,1.295)	0.48	0.57 (0.335,0.968)	0.037	0.92 (0.713,1.186)	0.518	1.034 (0.968,1.105)	0.324	0.858 (0.694,1.06)	0.156	1.003 (0.753,1.335)	0.986
MR-Egger	-	-	0.763 (0.335,1.737)	0.538	-	-	-	-	0.866 (0.587,1.279)	0.522	1.132 (0.67,1.911)	0.647
Weighted median	1.043 (0.830,1.310)	0.719	0.678 (0.347,1.326)	0.257	-	-	-	-	0.858 (0.665,1.108)	0.241	0.964 (0.627,1.482)	0.866
Penalised weighted median	1.043 (0.830,1.310)	0.718	0.686 (0.344,1.369)	0.285	-	-	-	-	0.858 (0.664,1.11)	0.244	0.964 (0.62,1.497)	0.869
MR-PRESSO	-	-	-	-	-	-	-	-	-	-	-	-
**Colon cancer**												
Maximum likelihood	0.999 (0.914,1.09)	0.973	0.771 (0.605,0.983)	0.036*	0.994 (0.888,1.113)	0.919	0.997 (0.968,1.026)	0.823	1.058 (0.955,1.172)	0.28	0.925 (0.811,1.055)	0.245
MR-Egger	-	-	0.811	0.448	-	-	-	-	0.865	0.27	1.225	0.253

			(0.486,1.355)						(0.702,1.068)		(0.872,1.72)	
Weighted median	0.987 (0.884,1.103)	0.822	0.699 (0.512,0.953)	0.024*	-	-	-	-	1.02 (0.9,1.155)	0.759	1.098 (0.902,1.338)	0.351
Penalised weighted median	0.987 (0.888,1.098)	0.814	0.699 (0.51,0.958)	0.026*	-	-	-	-	0.998 (0.872,1.142)	0.973	1.112 (0.919,1.346)	0.274
MR-PRESSO	-	-	-	-	-	-	-	-	-	-	0.981 (0.841,1.144)	0.808
**Pancreatic cancer**												
Maximum likelihood	1.147 (0.96,1.371)	0.132	1.174 (0.728,1.895)	0.51	0.869 (0.692,1.09)	0.224	0.97 (0.916,1.028)	0.306	0.885 (0.73,1.073)	0.213	1.122 (0.866,1.454)	0.384
MR-Egger	-	-	1.009 (0.429,2.374)	0.984	-	-	-	-	0.703 (0.494,1)	0.145	1.086 (0.656,1.797)	0.75
Weighted median	1.24 (0.988,1.556)	0.063	1.095 (0.6,1.997)	0.768	-	-	-	-	0.891 (0.691,1.149)	0.373	1.245 (0.846,1.831)	0.267
Penalised weighted median	1.253 (0.979,1.604)	0.073	1.09 (0.588,2.02)	0.784	-	-	-	-	0.891 (0.694,1.143)	0.364	1.245 (0.852,1.818)	0.258
MR-PRESSO	-	-	-	-	-	-	-	-	-	-	-	-
**Liver cancer**												
Maximum likelihood	0.974 (0.774,1.226)	0.822	1.426 (0.76,2.675)	0.269	0.884 (0.661,1.183)	0.408	0.987 (0.916,1.064)	0.738	0.895 (0.696,1.15)	0.385	1.19 (0.847,1.673)	0.316
MR-Egger	-	-	1.241 (0.383,4.018)	0.728	-	-	-	-	0.985 (0.621,1.564)	0.954	1.547 (0.829,2.885)	0.182
Weighted median	1.069 (0.798,1.434)	0.654	1.351 (0.637,2.863)	0.433	-	-	-	-	0.871 (0.641,1.185)	0.379	1.275 (0.771,2.109)	0.344
Penalised weighted median	1.069 (0.793,1.442)	0.66	1.35 (0.612,2.979)	0.457	-	-	-	-	0.871 (0.635,1.195)	0.392	1.278 (0.777,2.103)	0.334
MR-PRESSO	-	-	-	-	-	-	-	-	-	-	-	-
**FinnGen**												
**Esophageal cancer**												
Maximum likelihood	1.037 (0.721,1.493)	0.845	1.738 (0.605,4.99)	0.305	0.76 (0.441,1.311)	0.324	0.919 (0.815,1.036)	0.166	0.992 (0.654,1.505)	0.971	0.509 (0.297,0.872)	0.014

MR-Egger	-	-	2.161 (0.352,13.283)	0.43	-	-	-	-	1.249 (0.595,2.622)	0.599	0.215 (0.079,0.587)	0.006
Weighted median	1.09 (0.687,1.728)	0.714	2.264 (0.579,8.854)	0.24	-	-	-	-	1.087 (0.652,1.814)	0.748	0.381 (0.18,0.806)	0.012
Penalised weighted median	1.09 (0.692,1.717)	0.71	2.264 (0.557,9.211)	0.254	-	-	-	-	1.087 (0.658,1.798)	0.744	0.381 (0.178,0.815)	0.013
MR-PRESSO	-	-	-	-	-	-	-	-	-	-	-	
**Stomach cancer**												
Maximum likelihood	1.224 (0.935,1.603)	0.141	1.821 (0.937,3.539)	0.077	0.866 (0.653,1.148)	0.317	1.036 (0.964,1.113)	0.339	0.856 (0.657,1.116)	0.251	1.03 (0.743,1.428)	0.859
MR-Egger			0.963 (0.267,3.477)	0.956	-	-	-	-	0.975 (0.448,2.121)	0.953	0.802 (0.436,1.474)	0.484
Weighted median	1.209 (0.882,1.658)	0.238	1.536 (0.626,3.767)	0.348	-	-	-	-	0.971 (0.693,1.362)	0.866	0.9 (0.565,1.434)	0.658
Penalised weighted median	1.209 (0.873,1.676)	0.254	1.535 (0.657,3.586)	0.323	-	-	-	-	1.008 (0.736,1.381)	0.961	0.901 (0.576,1.412)	0.65
MR-PRESSO	-	-	-	-	-	-	-	-	-	-	-	
**Colon cancer**												
Maximum likelihood	0.944 (0.827,1.079)	0.398	0.737 (0.502,1.082)	0.119	1.107 (0.935,1.311)	0.239	0.958 (0.917,1)	0.052	0.907 (0.778,1.058)	0.213	0.841 (0.691,1.024)	0.085
MR-Egger			0.746 (0.386,1.44)	0.408	-	-	-	-	0.887 (0.678,1.16)	0.445	0.787 (0.519,1.195)	0.272
Weighted median	0.969 (0.826,1.136)	0.698	0.668 (0.396,1.127)	0.130	-	-	-	-	0.931 (0.777,1.114)	0.435	0.776 (0.58,1.039)	0.089
Penalised weighted median	0.969 (0.825,1.138)	0.701	0.668 (0.396,1.127)	0.131	-	-	-	-	0.931 (0.776,1.116)	0.439	0.776 (0.584,1.032)	0.081
MR-PRESSO	-	-	-	-	-	-	-	-	-	-	-	
**Pancreatic cancer**												
Maximum likelihood	0.942 (0.751,1.182)	0.607	0.898 (0.466,1.733)	0.749	0.705 (0.525,0.946)	0.020*	1.047 (0.974,1.127)	0.215	0.801 (0.62,1.036)	0.09	1.267 (0.907,1.77)	0.166

MR-Egger	-	-	0.754 (0.243,2.337)	0.638	-	-	-	-	0.668 (0.421,1.059)	0.184	1.668 (0.894,3.113)	0.121
Weighted median	0.947 (0.715,1.253)	0.701	1.097 (0.466,2.582)	0.833	-	-	-	-	0.75 (0.533,1.054)	0.098	1.292 (0.772,2.161)	0.329
Penalised weighted median	0.947 (0.714,1.256)	0.703	1.116 (0.485,2.566)	0.796	-	-	-	-	0.75 (0.539,1.043)	0.087	1.292 (0.787,2.12)	0.311
MR-PRESSO	-	-	-	-	-	-	-	-	-	-	-	
**Liver cancer**												
Maximum likelihood	1.011 (0.979,1.044)	0.501	0.338 (0.135,0.849)	0.021	0.937 (0.627,1.4)	0.749	1.072 (0.968,1.188)	0.181	0.92 (0.637,1.327)	0.654	1.453 (0.909,2.322)	0.118
MR-Egger			0.417 (0.058,2.977)	0.409	-	-	-	-	1.058 (0.447,2.506)	0.906	1.924 (0.756,4.898)	0.182
Weighted median	1.012 (0.972,1.053)	0.566	0.23 (0.066,0.795)	0.02	-	-	-	-	0.972 (0.629,1.503)	0.899	1.081 (0.521,2.246)	0.834
Penalised weighted median	1.012 (0.973,1.052)	0.552	0.22 (0.063,0.76)	0.017	-	-	-	-	0.972 (0.619,1.527)	0.903	1.08 (0.528,2.208)	0.834
MR-PRESSO	-	-	-	-	-	-	-	-	-	-	-	

The MR-Egger, weighted median and penalized weighted median require the number of SNPs in the instrumental variable > 2, and MR-Egger method cannot accurately estimate due to collinearity in MR analyses for α-tocopherols. The MR-PRESSO requires the number of SNPs in the instrument variable > 3. If the MR-PRESSO global test did not identify significant outliers in the genetic instrument, the MR-PRESSO did not require correction.

Table S6 The complementary MR analyses results of the causal effects of circulating antioxidant metabolites on five digestive system cancers.

MR methods	α-Tocopherol (11 SNPs)		γ-Tocopherol (13 SNPs)		Ascorbate (14 SNPs)		Retinol (26 SNPs)		Urate (18 SNPs)	
	OR (95%CI)	*p*-Value	OR (95%CI)	*p*-Value	OR (95%CI)	*p*-Value	OR (95%CI)	*p*-Value	OR (95%CI)	*p*-Value
**UK Biobank**										
**Esophageal cancer**										
Maximum likelihood	0.995 (0.846,1.17)	0.954	0.997 (0.989,1.005)	0.441	0.89 (0.653,1.212)	0.46	0.871 (0.784,0.968)	0.01	1.181 (0.945,1.477)	0.144
MR-Egger	0.854 (0.571,1.279)	0.464	0.987 (0.97,1.004)	0.167	1.208 (0.701,2.082)	0.51	0.865 (0.668,1.12)	0.28	1.405 (0.926,2.13)	0.129
Weighted median	0.995 (0.803,1.233)	0.966	0.999 (0.989,1.01)	0.875	0.967 (0.652,1.434)	0.867	0.848 (0.733,0.98)	0.026	1.129 (0.836,1.526)	0.429
Penalised weighted median	0.995 (0.8,1.239)	0.967	0.999 (0.988,1.01)	0.881	0.968 (0.655,1.429)	0.869	0.846 (0.738,0.97)	0.017	1.129 (0.843,1.512)	0.414
MR-PRESSO	-	-	-	-	-	-	-	-	-	-
Stomach cancer										
Maximum likelihood	1.072 (0.895,1.285)	0.45	0.924 (0.844,1.012)	0.09	0.921 (0.672,1.261)	0.606	0.937 (0.831,1.055)	0.282	1.024 (0.795,1.319)	0.855
MR-Egger	1.259 (0.824,1.925)	0.314	0.879 (0.722,1.069)	0.222	0.901 (0.521,1.56)	0.716	0.857 (0.617,1.19)	0.366	1.194 (0.651,2.19)	0.574
Weighted median	1.058 (0.839,1.334)	0.636	0.925 (0.817,1.047)	0.216	0.886 (0.578,1.358)	0.58	1.019 (0.868,1.196)	0.82	1.016 (0.724,1.425)	0.927
Penalised weighted median	1.058 (0.834,1.341)	0.644	0.925 (0.812,1.053)	0.237	0.886 (0.581,1.352)	0.575	1.021 (0.863,1.207)	0.812	1.018 (0.74,1.401)	0.913
MR-PRESSO	-	-	-	-	-	-	-	-	-	-
Colon cancer										
Maximum likelihood	1.001	0.972	1	0.92	0.928	0.327	1.025	0.382	0.994	0.276

	(0.922,1.088)		(0.996,1.004)		(0.799,1.078)		(0.97,1.082)		(0.982,1.005)	
MR-Egger	0.947 (0.78,1.15)	0.596	1.004 (0.994,1.014)	0.427	1.03 (0.801,1.324)	0.822	1.038 (0.89,1.211)	0.638	1.003 (0.981,1.024)	0.816
Weighted median	0.979 (0.875,1.096)	0.714	1.001 (0.996,1.007)	0.674	0.962 (0.791,1.17)	0.695	1.019 (0.941,1.103)	0.648	0.998 (0.983,1.013)	0.786
Penalised weighted median	0.979 (0.877,1.093)	0.708	1.001 (0.996,1.007)	0.653	0.962 (0.791,1.168)	0.693	1.015 (0.942,1.094)	0.695	0.998 (0.983,1.013)	0.786
MR-PRESSO	-	-	-	-	-	-	-	-	-	-
Pancreatic cancer										
Maximum likelihood	0.9 (0.763,1.061)	0.209	1.014 (0.933,1.102)	0.744	1.412 (1.045,1.908)	0.025	0.923 (0.83,1.027)	0.142	1.034 (0.824,1.296)	0.775
MR-Egger	0.605 (0.412,0.889)	0.031	0.901 (0.695,1.167)	0.445	1.535 (0.934,2.523)	0.117	1.003 (0.771,1.304)	0.983	1.013 (0.663,1.548)	0.952
Weighted median	0.865 (0.685,1.093)	0.224	0.985 (0.877,1.107)	0.805	1.438 (0.983,2.103)	0.061	0.935 (0.804,1.088)	0.387	1.036 (0.776,1.383)	0.812
Penalised weighted median	0.865 (0.688,1.088)	0.216	0.986 (0.875,1.112)	0.822	1.438 (0.951,2.173)	0.085	0.935 (0.811,1.079)	0.359	1.036 (0.763,1.406)	0.822
MR-PRESSO	-	-	1.039 (0.937,1.152)	0.473	-	-	-	-	-	-
Liver cancer										
Maximum likelihood	0.842 (0.68,1.043)	0.115	1.011 (0.908,1.125)	0.842	0.984 (0.676,1.432)	0.934	1.033 (0.896,1.191)	0.658	1.097 (0.812,1.481)	0.547
MR-Egger	0.638 (0.385,1.056)	0.114	0.95 (0.752,1.199)	0.674	0.976 (0.495,1.928)	0.946	0.775 (0.524,1.146)	0.213	1.155 (0.598,2.23)	0.673
Weighted median	0.79 (0.602,1.037)	0.089	1.023 (0.878,1.191)	0.772	0.92 (0.542,1.561)	0.758	1.118 (0.912,1.37)	0.282	1.133 (0.769,1.67)	0.526
Penalised weighted median	0.79 (0.602,1.037)	0.09	1.023 (0.882,1.185)	0.765	0.92 (0.559,1.514)	0.743	1.118 (0.919,1.359)	0.264	1.133 (0.776,1.656)	0.517
MR-PRESSO	-	-	-	-	-	-	-	-	-	-
FinnGen										
Esophageal cancer										

Maximum likelihood	1.186 (0.865,1.628)	0.289	1.097 (0.94,1.28)	0.24	0.863 (0.459,1.622)	0.646	0.987 (0.795,1.226)	0.909	0.874 (0.544,1.405)	0.579
MR-Egger	2.439 (1.089,5.461)	0.058	1.416 (1.024,1.958)	0.059	0.824 (0.192,3.534)	0.799	1.14 (0.679,1.914)	0.623	0.73 (0.282,1.892)	0.527
Weighted median	1.019 (0.65,1.598)	0.933	1.142 (0.927,1.407)	0.211	1.095 (0.48,2.494)	0.83	1.095 (0.812,1.476)	0.551	0.824 (0.438,1.551)	0.548
Penalised weighted median	1.019 (0.654,1.59)	0.932	1.142 (0.926,1.409)	0.215	1.099 (0.199,6.066)	0.914	1.095 (0.806,1.488)	0.562	0.824 (0.438,1.55)	0.548
MR-PRESSO	-	-	-	-	-	-	-	-	-	-
Stomach cancer										
Maximum likelihood	1.04 (0.86,1.259)	0.684	0.961 (0.873,1.057)	0.411	0.768 (0.525,1.122)	0.173	1.142 (1.003,1.302)	0.046	1.205 (0.906,1.603)	0.2
MR-Egger	1.009 (0.619,1.646)	0.971	1.111 (0.912,1.355)	0.319	0.658 (0.345,1.254)	0.227	1.015 (0.742,1.388)	0.928	1.236 (0.713,2.145)	0.462
Weighted median	1.02 (0.788,1.319)	0.882	0.976 (0.859,1.109)	0.709	0.768 (0.454,1.297)	0.323	1.154 (0.968,1.376)	0.11	1.225 (0.853,1.761)	0.272
Penalised weighted median	1.02 (0.791,1.314)	0.88	0.976 (0.855,1.114)	0.718	0.768 (0.419,1.405)	0.391	1.154 (0.966,1.379)	0.115	1.225 (0.832,1.803)	0.303
MR-PRESSO	-	-	-	-	-	-	-	-	-	-
Colon cancer										
Maximum likelihood	1.081 (0.961,1.215)	0.194	1.017 (0.96,1.077)	0.573	0.927 (0.735,1.169)	0.522	0.991 (0.915,1.072)	0.819	1.028 (0.864,1.222)	0.756
MR-Egger	1.112 (0.777,1.592)	0.574	1.044 (0.927,1.176)	0.493	1.02 (0.668,1.556)	0.929	1.078 (0.867,1.342)	0.504	1.448 (1.038,2.022)	0.045
Weighted median	1.025 (0.87,1.207)	0.771	0.984 (0.91,1.065)	0.692	1.022 (0.752,1.388)	0.89	1.031 (0.924,1.15)	0.587	1.123 (0.895,1.409)	0.317
Penalised weighted median	1.025 (0.864,1.214)	0.779	0.984 (0.91,1.063)	0.677	1.024 (0.761,1.379)	0.874	1.032 (0.924,1.153)	0.576	1.123 (0.889,1.418)	0.331
MR-PRESSO	-	-	-	-	-	-	-	-	-	-
Pancreatic cancer										
Maximum likelihood	1.114	0.289	1.01	0.844	0.876	0.498	1.022	0.749	1.117	0.461

	(0.912,1.36)		(0.917,1.112)		(0.596,1.286)		(0.894,1.169)		(0.833,1.497)	
MR-Egger	1.407	0.33	1.102	0.363	0.764	0.442	0.917	0.6	1.686	0.089
	(0.735,2.691)		(0.901,1.348)		(0.393,1.484)		(0.665,1.265)		(0.957,2.969)	
Weighted median	1.167	0.27	1.019	0.767	0.779	0.36	0.999	0.992	1.343	0.151
	(0.887,1.535)		(0.901,1.151)		(0.456,1.33)		(0.834,1.197)		(0.898,2.01)	
Penalised weighted	1.167	0.282	1.019	0.773	0.779	0.359	0.999	0.992	1.343	0.133
median	(0.881,1.545)		(0.898,1.155)		(0.457,1.328)		(0.831,1.202)		(0.914,1.975)	
MR-PRESSO	-	-	-	-	-	-	-	-	-	-
Liver cancer										
Maximum likelihood	0.992	0.956	1.036	0.612	0.666	0.139	0.898	0.267	1.251	0.287
	(0.755,1.305)		(0.904,1.187)		(0.388,1.141)		(0.743,1.086)		(0.828,1.891)	
MR-Egger	0.668	0.297	1.068	0.655	0.566	0.252	1.323	0.232	0.616	0.306
	(0.327,1.364)		(0.805,1.417)		(0.224,1.43)		(0.844,2.074)		(0.25,1.515)	
Weighted median	0.905	0.595	0.954	0.636	0.608	0.207	1.089	0.527	0.837	0.527
	(0.627,1.308)		(0.784,1.16)		(0.281,1.317)		(0.836,1.419)		(0.483,1.452)	
Penalised weighted	0.905	0.599	0.953	0.622	0.608	0.179	1.089	0.526	0.827	0.52
median	(0.624,1.312)		(0.786,1.154)		(0.294,1.257)		(0.836,1.419)		(0.464,1.474)	
MR-PRESSO	-	-	-	-	-	-	-	-	-	-

The MR-Egger, weighted median and penalized weighted median require the number of SNPs in the instrumental variable > 2. The MR-PRESSO requires the number of SNPs in the instrument variable > 3. If the MR-PRESSO global test did not identify significant outliers in the genetic instrument, the MR-PRESSO did not require correction.

Table S7 For the outcomes from other GWAS studies, the sensitivity MR analyses results of the causal effects of absolute ascorbate and retinol level on colon and pancreatic cancer.

Exposure	Outcome	MR methods	No. SNPs	Odds ratio (95%CI)	*p*-Value
**Ascorbate**	**Colon cancer (UKBB consortium, self-reported: colon cancer)**	**Main MR analysis**			
		Fixed-effect IVW	10	0.9993(0.9987,0.9999)	0.036
		**Complementary MR analysis**			
		Maximum likelihood	10	0.9993(0.9987,0.9999)	0.039
		MR-Egger	10	1.0000(0.9992,1.0009)	0.966
		Weighted median	10	0.9996(0.9989,1.0003)	0.271
		Penalised weighted median	10	0.9996(0.9989,1.0003)	0.270
		MR-PRESSO	10	-	-
**Retinol**	**Pancreatic cancer (PanScan1 consortium)**	**Main MR analysis**			
		Fixed-effect IVW	2	0.9630(0.7672,1.2088)	0.745
		**Complementary MR analysis**			
		Maximum likelihood	2	0.9621(0.7642,1.2112)	0.742
		MR-Egger	2	-	-
		Weighted median	2	-	-
		Penalised weighted median	2	-	-
		MR-PRESSO	2	-	-
